# PARP inhibitor BMN673 triggers PARylation-mediated ATF4-GDF15 pathway to drive autophagy and ferroptosis in ataxia telangiectasia mutated gene-deficient colorectal cancer cells

**DOI:** 10.1186/s43556-025-00356-6

**Published:** 2025-11-21

**Authors:** Junqi Xiang, Jie Xu, Hui Fan, Qian Chen, Yiting Lu, Xinyan Wan, Ying Jiang, Xia Zhang, Chundong Zhang, Qingyuan Liu, Degang Ding, Yunlong Lei

**Affiliations:** 1 Department of Biochemistry and Molecular Biology, and Molecular Medicine and Cancer Research Center, College of Basic Medical Sciences, Chongqing Medical University, Chongqing, 400016 P.R. China; 2 Tianfu Jincheng Laboratory, Chengdu, 610093 China; 3 The Center for Clinical Molecular Medical Detection, First Affiliated Hospital of Chongqing Medical University, Chongqing, 400016 China; 4 Department of Urology, Henan Provincial People’s Hospital, Zhengzhou, 450003 China

**Keywords:** PARP inhibitor BMN673, Colorectal cancer, Mitophagy, Ferroptosis, GDF15

## Abstract

Colorectal cancer (CRC) is a serious threat to human health, with an approximate 14% mutation rate in the ataxia telangiectasia-mutated (ATM) gene, which is involved in homologous recombination repair. BMN673 (talazoparib), a next-generation poly(ADP-ribose) polymerase (PARP) inhibitor, is the most potent PARP inhibitor (PARPi) reported to date, demonstrating robust anticancer activity. However, the precise mechanism underlying its action in ATM-deficient CRC remains unknown. This study demonstrated that BMN673 stimulated ATM-deficient CRC cell death via a synthetic lethal effect. RNA sequencing analysis revealed significant enrichment of the PERK-ATF4 pathway, mitophagy, and ferroptosis. Functional assays confirmed that BMN673 induced a multifaceted cell death program comprising autophagy-associated death, ferroptosis, and mitophagy, in addition to synthetic lethal. Mechanistically, BMN673 was shown to enhance activating transcription factor 4 (ATF4) transcriptional activity by suppressing poly-ADP-ribosylation (PARylation), facilitating ATF4 binding to the growth differentiation factor 15 (GDF15) promoter region and thereby inducing GDF15 transcriptional expression. Notably, GDF15 overexpression modulated the sensitivity of ATM-deficient CRC cells to BMN673 by promoting autophagy-associated cell death, ferroptosis, and mitophagy, contributing to the anticancer effect of BMN673. Additionally, combining BMN673 with radiotherapy exerted a synergistic anticancer effect on ATM-deficient CRC cells, which was prevented by autophagy inhibition. The findings identified the ATF4-GDF15 pathway as a crucial mediator of BMN673 sensitivity in ATM-deficient CRC cells, revealing therapeutic vulnerability beyond canonical DNA damage repair pathways and providing new insight for combination therapy strategies.

**Supplementary Information:**

The online version contains supplementary material available at 10.1186/s43556-025-00356-6.

## Introduction

Colorectal cancer (CRC) is a malignancy with high global incidence and mortality, ranking second and third among all cancers worldwide, respectively [1–3]. GLOBOCAN 2024 estimated over 2 million new CRC cases and 1.1 million deaths, with estimated projections of up to 3.2 million new cases and 1.6 million deaths by 2044 [4]. Despite advancements in standard therapies, treatment outcomes remain suboptimal due to poor sensitivity and drug resistance. Targeted therapies have emerged as promising approaches, offering advantages such as specificity and reduced side effects [5–7].

Poly(ADP-ribose) polymerase (PARP) inhibitors have garnered significant attention due to their ability to induce synthetic lethality in tumor cells with homologous recombination repair (HRR) dysfunction, such as mutations in *BRCA1*, *BRCA2*, or other HRR-related genes like ataxia telangiectasia-mutated (*ATM*) [8, 9]. The clinical significance of PARP inhibition is underscored by its approval by regulatory authorities for treating various cancer types, including ovarian, breast, and pancreatic cancers [10]. BMN673 (talazoparib) stands out due to its superior PARP-trapping capability, demonstrating 20- to 200-fold greater potency than earlier generation inhibitors in preclinical models [11]. Although ATM mutations are present in approximately 14% of CRC patients, the precise mechanisms of action of PARP inhibitors, particularly BMN673, in ATM-deficient CRC are not fully elucidated, especially the impact of potent PARP-trapping on DNA repair pathway rewiring [12].

Beyond direct DNA damage, PARP inhibitors engage other cell death pathways. Autophagy, a process with dual roles in cancer, can be significantly induced in various cancers by treatment with PARP inhibitors, often augmenting the anticancer effects of autophagy [13–17]. PARP inhibitors can also engage multiple non-apoptotic cell death pathways beyond their direct DNA-damaging effects, including ferroptosis [18, 19]. Ferroptosis is an iron-dependent form of regulated cell death and has emerged as a novel therapeutic target in CRC [20]. Although initially not considered a direct consequence of DNA damage repair, recent findings have illuminated the capacity of PARP inhibitors to induce ferroptosis, either in synergy with other agents or under specific therapeutic regimens [21, 22]. Moreover, endoplasmic reticulum (ER) stress and autophagy are stress response mechanisms that cooperate to maintain cellular homeostasis. Activating transcription factor 4 (ATF4), a central mediator of ER stress, governs adaptive gene expression [23, 24]. PARP inhibitors have been linked to ER stress, and glutathione peroxidase 4 (GPX4), a key ferroptosis regulator, was reported to be upregulated post-PARP inhibitor treatment in an ATF4-dependent manner [25–27], suggesting potential crosstalk.

This study discovered that BMN673 exerts its anticancer activity against ATM-deficient CRC cells primarily through a synthetic lethal mechanism. Notably, the findings extend beyond this basic mechanism, revealing that BMN673 further enhances its therapeutic effect by eliciting mitochondrial dysfunction and orchestrating a cascade of cellular processes, specifically autophagy-associated cell death, ferroptosis, and mitophagy. This orchestrated response is facilitated by the ER stress-related ATF4-GDF15 signaling axis, underscoring the intricate interplay between these pathways. Moreover, BMN673 was found to markedly potentiate the sensitivity of ATM-deficient CRC cells to radiation therapy (RT), an effect that was attenuated upon autophagy inhibition.

## Results

### PERK-ATF4 signaling pathway, mitophagy, and ferroptosis may be involved in the anticancer effect of BMN673 in ATM-deficient colorectal cancer cells

**Fig. 1:**
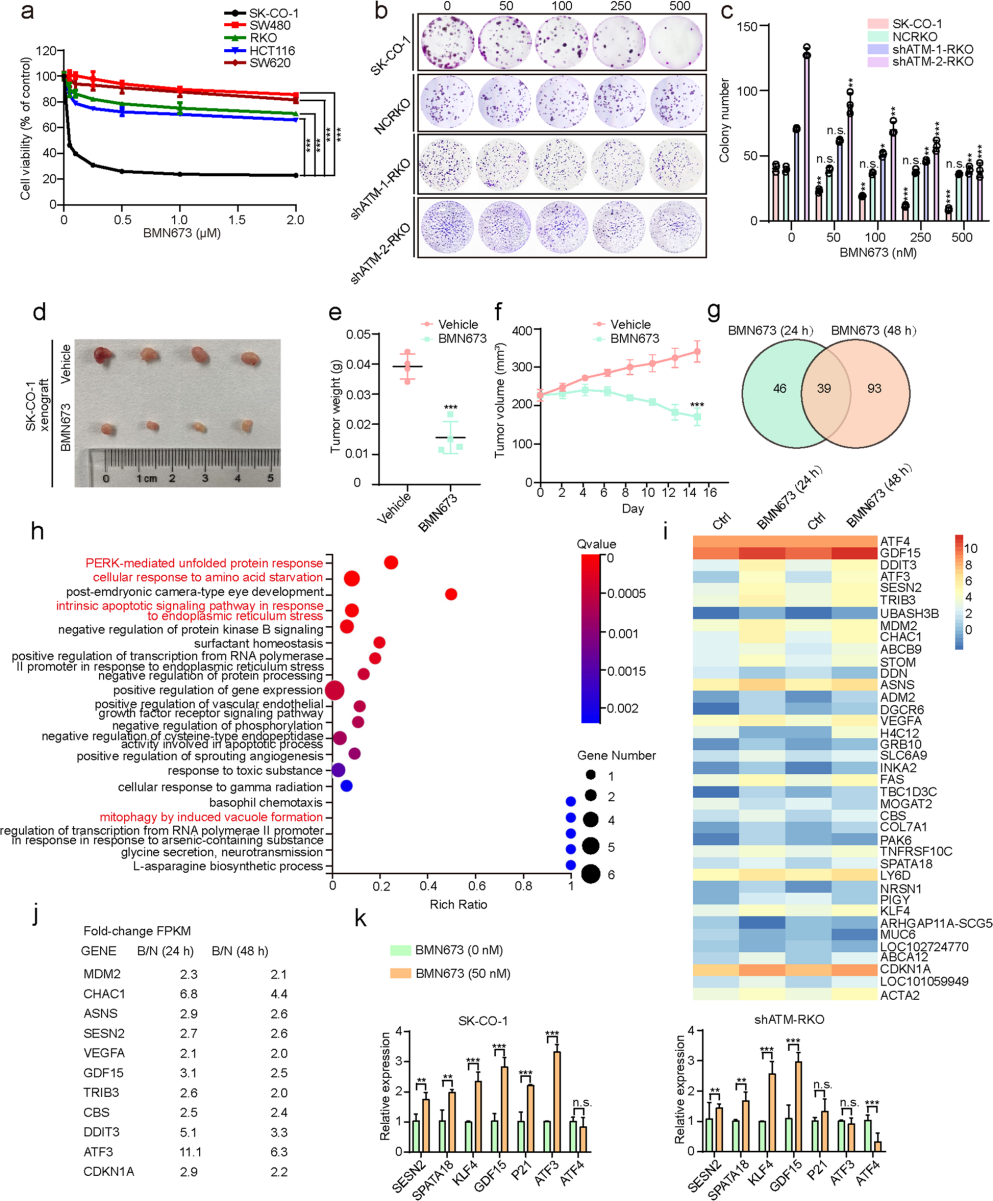
PERK-ATF4 signaling pathway, mitophagy, and ferroptosis may be involved in the anticancer effect of BMN673 in ATM-deficient colorectal cancer cells. **a** CCK8 assay of SK-CO-1, SW480, RKO, HCT-116 and SW620 cells treated with the indicated concentrations of BMN673 for 48 h. **b**, **c** Colony formation assay of SK-CO-1, non-targeting control (NC) RKO and shATM-RKO cells treated with the indicated concentrations of BMN673 for 2 weeks (**b**), and quantification of colonies (**c**) were shown. **d**-**f** A subcutaneous xenograft model was established in nude mice using SK-CO-1 cells. The tumor-bearing mice were then treated with BMN673 via oral gavage. Tumor size was measured every two days to plot the growth curves. At the endpoint, the tumors were harvested and their final volume and weight were measured. Images (**d**) and weights (**e**) of isolated tumors, and tumor volumes monitored at the indicated time points (**f**). **g**-**j** Differentially expressed genes were examined by RNA-seq in SK-CO-1 cells treated with 50 nM BMN673 for 24 and 48 h. **g** Venn diagram showed that 39 genes are differentially expressed in both 24 h treatment group (left) and 48 h treatment group (right); (**h**) Gene ontology (GO) enrichment; (**i**) Differences in gene heat maps; (**j**) Differentially expressed genes involved in the regulation of ferroptosis. (**k**) Quantitative PCR to verify RNA-seq results in SK-CO-1 and shATM-RKO cells treated with 50 nM BMN673 for 48 h. Data are means ± s.d. and are representative of 3 independent experiments. *, *P* < 0.05, **, *P* < 0.01, ***, *P* < 0.001

The CCK8 assay was used to quantify the viability of various human CRC cell lines (SK-CO-1, SW480, RKO, HCT116, and SW620) treated with BMN673 and substantiate the antitumor efficacy of BMN673 in ATM-deficient CRC. Notably, SK-CO-1 cells, with ATM deficiency (Fig. S1a), demonstrated exceptional sensitivity to BMN673, whereas other CRC cell lines exhibited significant tolerance (Fig. 1a). The specific role of ATM deficiency in mediating sensitivity to BMN673 was investigated by pharmacologically inhibiting ATM in three ATM-proficient cell lines (HT29, RKO, and HCT116) using the ATM inhibitor KU-55933 (Fig. S1b). While treatment with either BMN673 or KU-55933 alone did not significantly affect cell viability, the combination of BMN673 and KU-55933 markedly decreased cell viability in all three cell lines (Fig. S1c). Furthermore, SK-CO-1 cells overexpressing ATM (Fig. S1d) significantly rescued decreases in cell viability following BMN673 treatment compared to the control group (Fig. S1e). Two ATM-knockdown RKO cell lines were generated using lentiviral expression to further validate the cytotoxic effects of BMN673 associated with ATM deficiency (Fig. S1f). These knockdown RKO lines exhibited reduced cell growth following BMN673 treatment compared to the controls, suggesting that ATM deficiency confers sensitivity to this PARP inhibitor (Fig. 1b-c and S1g). Subsequent experiments used the shATM-2 group, with better knockout efficiency. Next, an ATM mutant plasmid (ATM^*D1853N*^) was constructed and introduced into SK-CO-1 cells using a lentiviral system to validate the findings in a model more closely resembling the ATM mutations found in clinical CRC patients [28, 29]. The mutant group exhibited a concentration-dependent decrease in cell viability following the introduction of the ATM mutation compared to the wild-type control group (Fig. S1h). This finding was reinforced by dose-dependent decreases in EdU incorporation, accompanied by a modest increase in PARP cleavage, in BMN673-treated ATM-deficient CRC cells (Fig. S1i-k). Flow cytometry analysis revealed a G2/M phase arrest in these cells (Fig. S1l). Thus, these data indicate that BMN673 inhibits ATM-deficient CRC cell growth by inhibiting cell cycle progression and inducing apoptosis in vitro.

The antitumor effects of BMN673 against ATM-deficient CRC cells were confirmed in vivo by employing a subcutaneous xenograft model in nude mice, wherein BMN673 administration significantly inhibited tumor growth, as evidenced by reduced tumor volumes and weights (Fig. 1d-f). Immunohistochemical analysis corroborated these findings, demonstrating enhanced cleaved caspase-3 (CC3) staining and decreased Ki67 expression in BMN673-treated tumors (Fig. S1m-o). Taken together, these data indicate that BMN673 inhibits ATM-deficient CRC cell growth both in vitro and in vivo.

RNA sequencing technology was employed to analyze differentially expressed genes after 24 h and 48 h of BMN673 treatment and delve deeper into the anticancer mechanisms of BMN673 beyond its synthetic lethal effect in ATM-deficient CRC cells. The number of significantly altered genes was relatively modest, with 85 genes differentially expressed at 24 h and 132 at 48 h, sharing only 39 common variations (Fig. 1g and Supplementary Table 1). Gene Ontology (GO) enrichment analysis highlighted the prominent enrichment of the PERK-ATF4 signaling pathway and the mitophagy signaling pathway, both of which are intimately linked to cancer resistance (Fig. 1h). The heat map visualization further confirmed that classical ATF4 target genes were markedly upregulated (Fig. 1i), underscoring the activation of this regulatory axis. Moreover, cross-analysis with the ferroptosis database (FerrDb website (http://www.zhounan.org/ferrdb/)) revealed that nearly one-third of the differentially expressed genes were involved in ferroptosis regulation (Fig. 1j). Given the pivotal roles of the PERK-ATF4 ER stress signaling pathway, autophagy, and ferroptosis in anticancer therapies [30], this finding underscores the complex interplay of these mechanisms in BMN673’s antitumor effects. The expression levels of the differentially regulated genes in both SK-CO-1 and shATM-RKO cells were analyzed to validate these observations. While the majority of the PERK-ATF4 target genes were upregulated upon BMN673 treatment, the transcription of ATF4 itself was downregulated (Fig. 1k). This paradoxical finding implies that additional, but as yet unidentified mechanisms might be involved, orchestrating the activation of the PERK-ATF4 signaling pathway in response to BMN673.

### Autophagy potentiates BMN673-induced anticancer effects

**Fig. 2:**
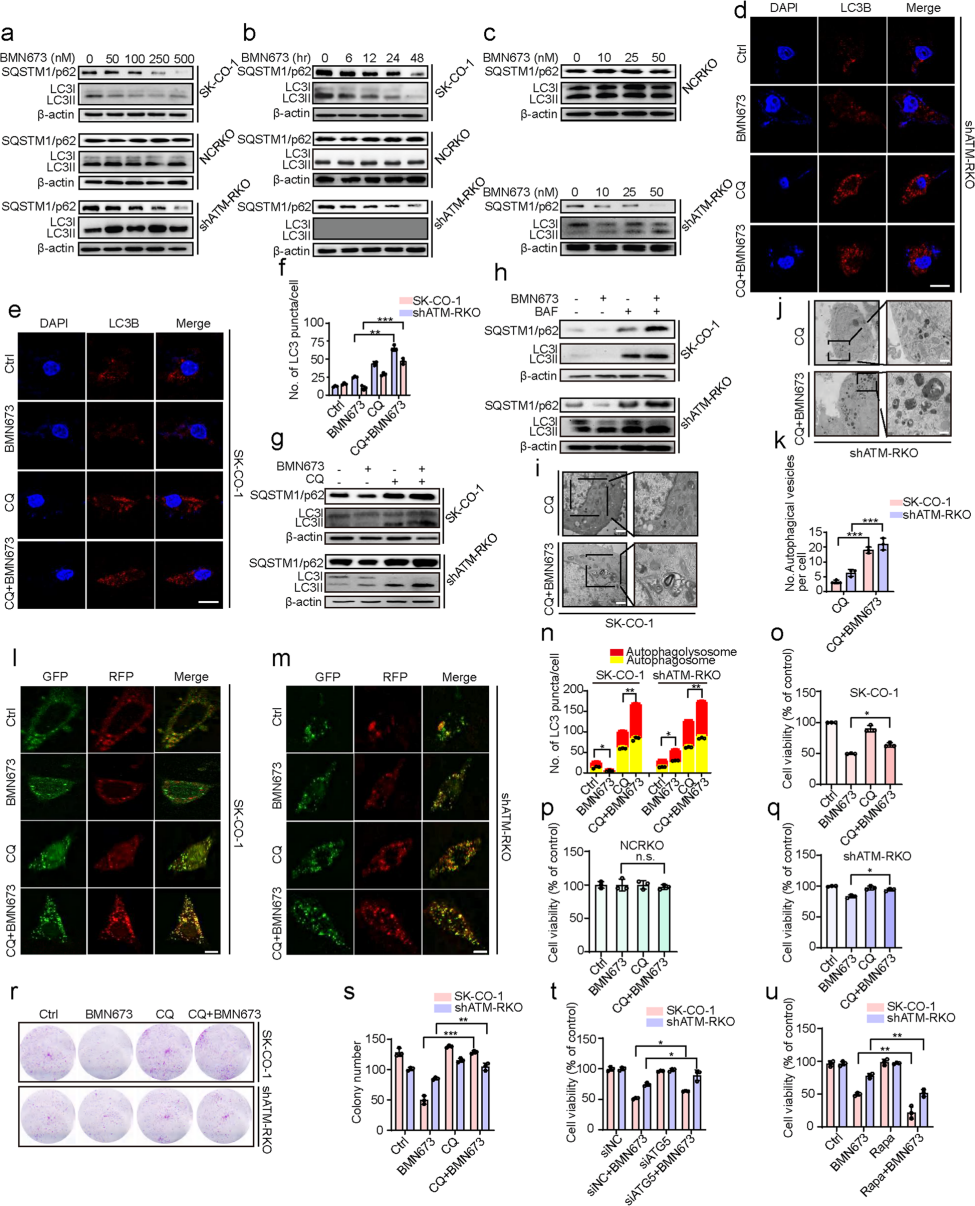
Autophagy potentiates BMN673-induced anticancer effects. **a** Immunoblot analysis of LC3B and SQSTM1/p62 expression in SK-CO-1, NCRKO and shATM-RKO cells treated with the indicated concentrations of BMN673 for 48 h. **b** Time course analysis of LC3B and SQSTM1/p62 expression in SK-CO-1, NCRKO and shATM-RKO cells treated with 50 nM BMN673 for 6, 12, 24, 48 h by immunoblotting. **c** Immunoblot analysis of LC3B and SQSTM1/p62 expression in NCRKO and shATM-RKO cells treated with the indicated concentrations of BMN673 for 48 h. **d**, **e** Immunofluorescence analysis of LC3 in ATM-deficient colorectal cancer cells treated with BMN673 (50 nM) alone or in combination with 10 μM CQ for 48 h. And the number of LC3 puncta (**f**) was quantitated. Scale bar, 20 μm. **g**, **h** SK-CO-1 and shATM-RKO were treated with BMN673 (50 nM) alone or in combination with 10 μM CQ or 100 nM BAF for 48 h. The expressions of LC3B and SQSTM1/p62 were examined by immunoblotting. **i**-**k** Autophagic vesicles were detected by transmission electron microscope in SK-CO-1 and shATM-RKO cells treated with or without 50 nM BMN673 in combination with 10 μM CQ for 48 h. Scale bar, 1 μm. (**l**, **m**) Immunofluorescence analysis of cells transiently transfected with tandem mRFP-GFP-tagged LC3B and treated with 50 nM BMN673, 10 μM CQ, or in combination for 48 h. Scale bar, 10 μm, and the ratio of red puncta indicating autolysosomes versus yellow puncta indicating autophagosomes (**n**) was quantitated. GFP: GFP-LC3B; RFP: RFP-LC3B. (**o-q**) SK-CO-1 (**o**), NCRKO (**p**) and shATM-RKO cells (**q**) were treated with BMN673 (50 nM) alone or in combination with 10 μM CQ for 48 h. The cell viability was examined by CCK8 assay. (**r**) Cell proliferation rate was analyzed by clone formation assay. SK-CO-1 and shATM-RKO cells were treated with 50 nM BMN673 in the presence or absence of 10 μM CQ for 48 h, after treatment, cells were seeded into 12-well plates for two weeks and colony numbers (**s**) were quantified. (**t**) SK-CO-1 and shATM-RKO cells transfected with siNC or siATG5 for 24 h, followed by treatment with or without 50 nM BMN673 for another 48 h. The cell viability was examined by CCK8 assay. (**u**) SK-CO-1 and shATM-RKO cells were treated with BMN673 (50 nM) alone or in combination with 10 μM Rapa for 48 h. The cell viability was examined by CCK8 assay. Data are means ± s.d. and are representative of 3 independent experiments. *, *P* < 0.05, **, *P* < 0.01, ***, *P* < 0.001

The RNA-sequencing results highlighted the crucial role of autophagy in the response of ATM-deficient CRC cells to BMN673 treatment. This finding was validated by first examining whether BMN673 could regulate autophagy in ATM-deficient CRC cells. BMN673 treatment inhibited the accumulation of LC3Ⅱ in SK-CO-1 cells, but promoted the accumulation of LC3Ⅱ in ATM stably knocked-down RKO cells, while SQSTM1/p62 was reduced in both cell lines in a dose- and time-dependent manner (Fig. 2a, b). Notably, autophagic vacuole accumulation was altered by treatment with a low dose of BMN673 (50 nM) in SK-CO-1 cells and RKO cells with the stable knockdown of ATM. Therefore, ATM stably knocked down RKO cells were treated with BMN673 (0–50 nM). A very low dose of BMN673 (10 nM) promoted the accumulation of LC3Ⅱ and reduced SQSTM1/p62 expression but not in their control counterparts (Fig. 2c). The autophagic phenotype was further supported by changes in autophagic vesicles (LC3B puncta) in the BMN673 and chloroquine (CQ) combination treatment groups compared with BMN673-treated groups (Fig. 2d-f). Immunohistochemical analysis of tumor tissue from the xenograft model similarly revealed weaker LC3 staining in the BMN673-treated SK-CO-1 groups compared to the controls (Fig. S2a, b), suggesting a change in autophagy flux.

LC3II changes are likely to be affected by autophagy initiation but may also be regulated by autophagic flux. Therefore, bafilomycin A1 (BAF), which inhibits autophagic flux by inhibiting the acidity of the autolysosome environment, and CQ, an autolysosome binding inhibitor, were used in combination with BMN673. Treatment with BMN673 combined with CQ or BAF led to a further increase in the accumulation of LC3II and SQSTM1/p62 in both SK-CO-1 and shATM-RKO cells compared with single treatments alone, suggesting that BMN673 could promote complete autophagic flux in ATM-deficient CRC cells (Fig. 2g, h and Fig. S2c). Transmission electron microscopy further corroborated these findings, revealing an increase in autophagosomes upon combined treatment with BMN673 and CQ (Fig. 2i-k and Fig. S2d, e). Tandem monomeric RFP-GFP-tagged LC3B was used to monitor autophagy flux to gain a more dynamic view of autophagy progression. The result showed that BMN673 treatment increased both yellow autophagosomes and red fluorescent autolysosomes in shATM-RKO cells but decreased these in SK-CO-1 cells. Yellow fluorescent autophagosomes were significantly increased in groups co-treated with BMN673 and CQ compared with groups treated with BMN673 or CQ alone (Fig. 2l-n). These comprehensive findings firmly establish that BMN673 promotes robust and complete autophagic flux in ATM-deficient CRC cells.

The autophagy inhibitor CQ was used to inhibit autophagy and elucidate the role of autophagy in the anticancer activity of BMN673 in ATM-deficient CRC cells. The therapeutic response was subsequently assessed using CCK8 assays. Co-treatment with CQ notably rescued the viability of ATM-deficient CRC cells compared to BMN673 treatment alone (Fig. 2o-q). This observation was further substantiated using colony formation assays (Fig. 2r, s and Fig. S2f, g) and EdU labeling (Fig. S2h-k), which collectively indicated that autophagy inhibition counteracted the cytotoxic effects of BMN673. The role of autophagy was independently confirmed by knocking down ATG5, a key autophagic protein (Fig. S2l), to provide genetic evidence. Consistently, ATG5 knockdown significantly mitigated the anticancer impact of BMN673, as evidenced by increased cell survival rates in ATM-deficient CRC cells (Fig. 2t). The autophagy inducer rapamycin (Rapa) was used to induce autophagy. The CCK8 assay and colony formation assay revealed that co-treatment with Rapa significantly reduced the viability of ATM-deficient CRC cells compared with BMN673 treatment alone (Fig. 2u and Fig. S2m, n). Collectively, these findings underscore the pivotal role of autophagy in amplifying the anticancer effects of BMN673 in ATM-deficient CRC cells, reinforcing the notion that autophagy represents a crucial mediator of BMN673’s therapeutic efficacy.

### BMN673 induces ferroptosis and disrupts mitochondrial function in ATM-deficient colorectal cancer cells

**Fig. 3:**
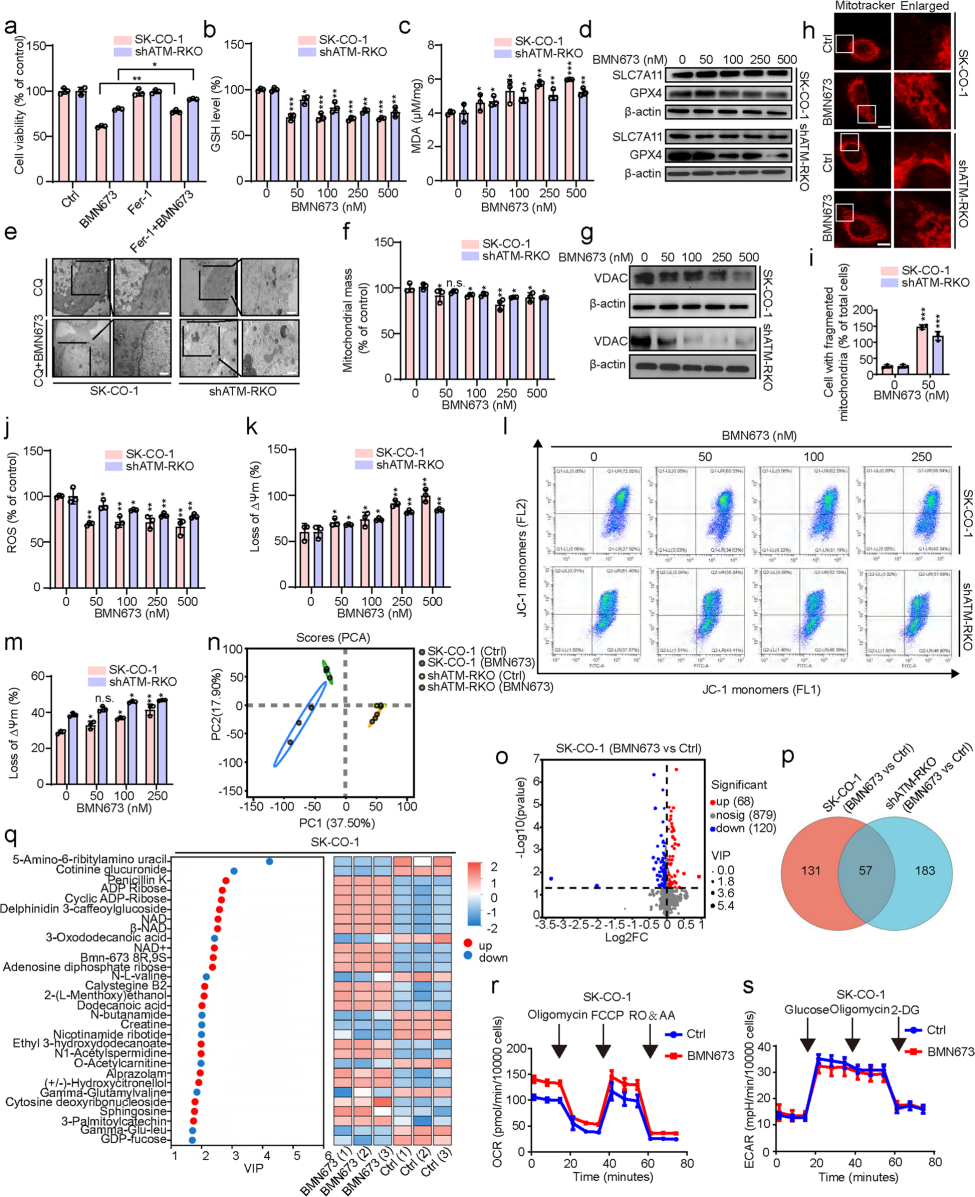
BMN673 induces ferroptosis and disrupts mitochondrial function in ATM-deficient colorectal cancer cells. **a** SK-CO-1 and shATM-RKO cells were treated with 50 nM BMN673 alone or in combination with 1 μM Fer-1 for 48 h. The cell viability was examined by CCK8 assay. **b**, **c** GSH level (**b**) and MDA content (**c**) in SK-CO-1 and shATM-RKO cells following indicated concentrations of BMN673 treatment for 48 h were determined. **d** Immunoblot analysis of SLC7A11 and GPX4 expression in SK-CO-1 and shATM-RKO cells treated with the indicated concentrations of BMN673 for 48 h. **e** Mitochondrial morphology was detected by transmission electron microscope in SK-CO-1 and shATM-RKO cells treated with or without 50 nM BMN673 in combination with 10 μM CQ for 48 h. Scale bar, 1 μm. **f**, **g** Mitochondrial mass were analyzed by Mito-Tracker Green staining (**f**) and immunoblotting of VDAC (**g**) in SK-CO-1 and shATM-RKO cells treated with the indicated concentrations of BMN673. **h** Mito-Tracker Deep Red FM staining SK-CO-1 and shATM-RKO cells treated with or without 50 nM BMN673 for 48 h and fragment mitochondria (**i**) were quantified. Scale bar, 10 μm. **j** ROS levels were analyzed by DCFH-DA staining in cells treated with indicated concentrations of BMN673. (k and l, m) Mitochondrial membrane potential changes were detected by JC-1 staining and analyzed by microplate reader (**k**) and flow cytometric analysis (**l**, **m**) in SK-CO-1 and shATM-RKO cells treated with indicated concentrations of BMN673. **n**-**q** Differential metabolites were examined by metabolomics analysis in SK-CO-1 cells and shATM-RKO cells treated with 50 nM BMN673 for 48 h. **n** PCA of untargeted metabolomics data from BMN673-treated SK-CO-1 cells and shATM-RKO cells. **o** Volcano plot of all DAMs. **p** Venn diagram showed that 57 differential metabolites are changed in both BMN673-treated SK-CO-1 cells group (left) and BMN673-treated shATM-RKO cells group (right); (**q**) VIP analysis. **r**, **s** OCR and ECAR in SK-CO-1 cells treated with or without 50 nM BMN673 was measured at 48 h. Data are means ± s.d. and are representative of 3 independent experiments. *, *P* < 0.05, **, *P* < 0.01, ***, *P* < 0.001

Ferrostatin-1 (Fer-1), a ferroptosis inhibitor, was used in combination with BMN673 to evaluate whether BMN673 induces ferroptosis in ATM-deficient CRC. Fer-1 significantly prevented the anticancer effect of BMN673 in ATM-deficient CRC cells (Fig. 3a and Fig. S3a), suggesting that BMN673 could induce ferroptosis in ATM-deficient CRC. Further validation was performed by examining ferroptosis markers, including glutathione (GSH) levels, lipid oxidation, and the expression of solute carrier family 7 member 11 (SLC7A11) and GPX4. The results revealed that BMN673 treatment markedly downregulated GSH levels and GPX4 expression while promoting lipid oxidation in ATM-deficient CRC cells (Fig. 3b-d and Fig. S3b-d). Immunohistochemical analysis of tumor tissue from the xenograft model similarly revealed weaker GPX4 staining in BMN673-treated SK-CO-1 groups compared to controls (Fig. S3e, f). Furthermore, GPX4 knockdown in the combination treatment groups led to a further reduction in cell viability compared to the groups treated with the combination of Fer-1 and BMN673 (Fig. S3g, h). The knockdown of GPX4 in both SK-CO-1 and shATM-RKO cells exacerbated BMN673-induced GSH depletion and malondialdehyde (MDA) accumulation (Fig. S3i, j). Morphologically, ferroptotic cells are characterized by shrunken mitochondria and increased membrane density, distinguishable from other forms of cell death [31]. Consistently, denser mitochondrial morphology was observed in BMN673-treated ATM-deficient CRC cells (Fig. 3e).

The morphology, quantity, and function of mitochondria were evaluated to ascertain the mitochondrial alterations in response to BMN673 treatment in ATM-deficient CRC cells. Notably, a mild decrease in mitochondrial mass, as determined by Mito-Tracker Green staining and immunoblotting for the mitochondrial component voltage-dependent anion channel (VDAC), was seen (Fig. 3f, g). BMN673 treatment caused a shift from tubular mitochondria to ring-shaped structures (Fig. 3h, i), indicative of mitochondrial shrinkage or fragmentation. Immunohistochemical analysis also confirmed attenuated VDAC staining in xenograft tissues post-BMN673 treatment (Fig. S3k, l). Along with decreases in mitochondrial quantity, changes in mitochondrial function were also examined by evaluating the mitochondrial membrane potential and mitochondrial reactive oxygen species (ROS) generation. Consistently, BMN673-treated cells exhibited a decline in mitochondrial membrane potential; however, BMN673-treated cells exhibited decreased ROS generation, potentially linked to enhanced mitophagy (Fig. 3j-m). A metabolomics analysis was conducted to further investigate whether BMN673 treatment induced mitochondrial quality control in ATM-deficient CRC cells. After 48 h of BMN673 treatment, principal component analysis confirmed metabolic separation between SK-CO-1 cells and shATM-RKO cells (Fig. 3n). 188 and 240 differential accumulation metabolites were identified in SK-CO-1 and shATM-RKO cells, respectively. Of these, 120 were downregulated and 68 were upregulated in SK-CO-1 cells, while 21 were downregulated and 219 were upregulated in shATM-RKO cells (Fig. 3o and Fig. S3m), with 57 metabolites common across both cell lines (Fig. 3p and Supplementary Table 2). Kyoto Encyclopedia of Genes and Genomes enrichment analysis revealed that these common differential metabolites were primarily enriched in pathways related to mitochondrial function, ER stress, and ferroptosis, including oxidative phosphorylation, calcium signaling, and sphingolipid signaling pathways (Fig. S3n). Further variable importance in projection (VIP) analysis of the differential metabolites in BMN673-treated SK-CO-1 and shATM-RKO cells identified NAD, NAD^+^, and ADP ribose as significantly contributing metabolites, all of which were upregulated (Fig. 3q and Fig. S3o). Moreover, oxygen consumption rate (OCR) and extracellular acidification rate (ECAR), key indicators of cellular energy metabolism, were assessed. The data indicated that BMN673 treatment enhanced oxidative phosphorylation and reduced glycolysis in both SK-CO-1 and shATM-RKO cells (Fig. 3r, s and Fig. S3p, q). These findings suggest that mitochondrial dysfunction may trigger mitochondrial quality control mechanisms, including mitophagy, and also indicate the potential involvement of ER stress and ferroptosis. Taken together, these data indicate that BMN673 induced ferroptosis, mitochondrial loss, and mitochondrial function impairment in ATM-deficient CRC cells.

### BMN673 promotes mitophagy in ATM-deficient colorectal cancer cells

**Fig. 4:**
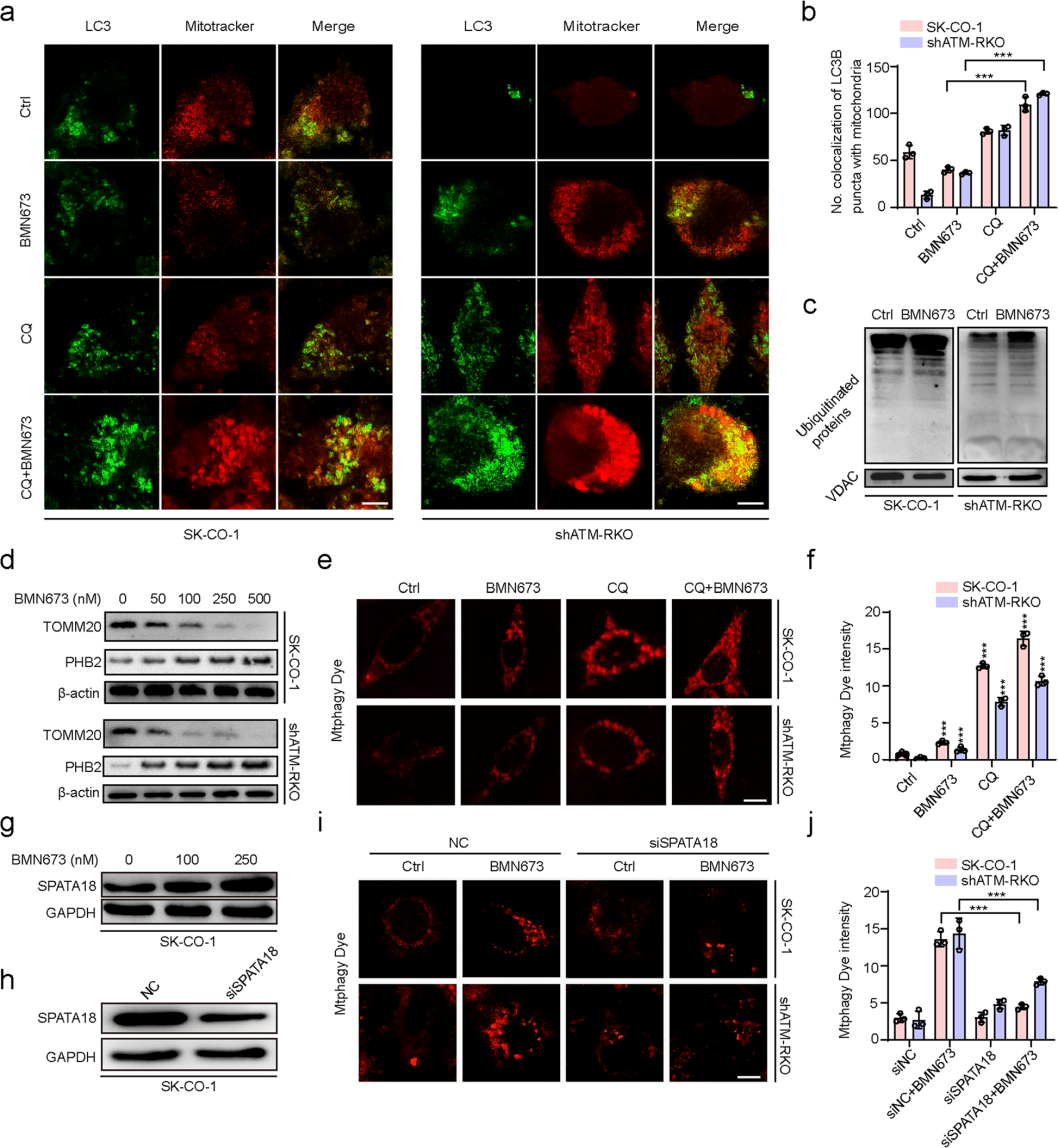
BMN673 promotes mitophagy in ATM-deficient colorectal cancer cells. **a**, **b** Immunofluorescence analysis of the co-localization of endogenous LC3B and Mitotracker in SK-CO-1 and shATM-RKO cells treated with vehicle, BMN673 (50 nM), CQ (10 μM), or in combination for 48 h. Scale bar, 10 μm. **c** Immunoblotting of ubiquitinated proteins in the mitochondrial fractions from SK-CO-1 and shATM-RKO cells treated with or without 50 nM BMN673 for 48 h. **d** Immunoblot analysis of TOMM20 and PHB2 expression in SK-CO-1 and shATM-RKO cells treated with the indicated concentrations of BMN673 for 48 h. **e**, **f** SK-CO-1 and shATM-RKO cells were treated with vehicle, BMN673 (50 nM), CQ (10 μM), or in combination for 48 h. Then the cells were treated with Mtphagy Dye, a mitophagy detection reagent, for 30 min. Scale bar, 10 μm. **g** Immunoblot analysis of SPATA18 expression in SK-CO-1 cells treated with the indicated concentrations of BMN673 for 48 h. **h** Immunoblot analysis of SPATA18 expression in SK-CO-1 cells transfected with siNC or siSPATA18 for 24 h. **i**, **j** SK-CO-1 and shATM-RKO cells were transfected with siNC or siSPATA18 for 24 h, followed by treatment with or without 50 nM BMN673 for another 24 h. Then the cells were treated with Mtphagy Dye, a mitophagy detection reagent, for 30 min. Scale bar, 10 μm. Data are means ± s.d. and are representative of 3 independent experiments. ***, *P* < 0.001

Mitophagy, a quality control mechanism that eliminates dysfunctional mitochondria, plays a pivotal role in maintaining cellular homeostasis [32]. Given the prior findings indicating BMN673-induced autophagy flux and mitochondrial dysfunction in ATM-deficient CRC cells, whether BMN673 induced mitophagy in ATM-deficient CRC cells was investigated. Immunofluorescence staining for LC3B was performed in combination with Mitotracker or ATP5B (a marker for mitochondria). The results showed that BMN673 treatment decreased mitochondria-LC3B colocalization in SK-CO-1 cells but increased this colocalization in shATM-RKO cells, while mitochondria-LC3B colocalization was significantly increased in groups co-treated with BMN673 and CQ compared to CQ alone-treated groups (Fig. 4a, b and Fig. S4a, b). Furthermore, the ubiquitination of mitochondrial proteins, a hallmark of mitophagy induction, was elevated in BMN673-treated cells (Fig. 4c). In addition, treatment of both SK-CO-1 and shATM-RKO cells with BMN673 led to a dose-dependent decrease in the abundance of translocase of outer mitochondrial membrane 20 (TOMM20), a mitochondrial outer membrane protein. Concurrently, the level of prohibitin 2 (PHB2), a mitochondrial inner membrane protein that functions as a mitophagy receptor, was dose-dependently increased (Fig. 4d and Fig. S4c). In addition, ATG5 knockdown restored TOMM20 expression and suppressed PHB2 increases (Fig. S4d). The Mtphagy Detection Kit was used to visually confirm the induction of mitophagy in response to BMN673 and provide direct evidence of mitophagy. Co-treatment with CQ, an inhibitor of autophagosome-lysosome fusion, further augmented mitophagy (Fig. 4e, f), suggesting that alternative pathways independent of canonical autophagic machinery may also contribute to this process. The RNA-sequencing data revealed an upregulation of SPATA18, a p53-inducible protein, which is implicated in DNA damage-induced mitophagy [33]. SPATA18 expression was elevated in SK-CO-1 cells (Fig. 4g), while the knockdown of SPATA18 significantly attenuated BMN673-mediated mitophagy (Fig. 4h-j), reinforcing its role in regulating this process. Furthermore, functional assays revealed that SPATA18 downregulation partially restored cell proliferation and viability in BMN673-treated ATM-deficient CRC cells (Fig. S4e-g), underscoring the significance of mitophagy in mediating the antitumor effects of BMN673. Collectively, these findings indicate the ability of BMN673 to promote both LC3-mediated conventional mitophagy and SPATA18-dependent mitophagy in ATM-deficient CRC cells. This dual-pronged approach to mitochondrial quality control represents a novel mechanism by which BMN673 exerts its antitumor effects, potentially expanding the therapeutic utility of PARP inhibitors in this cancer subtype.

### ATF4-GDF15 axis contributes to the anticancer effects of BMN673

**Fig. 5:**
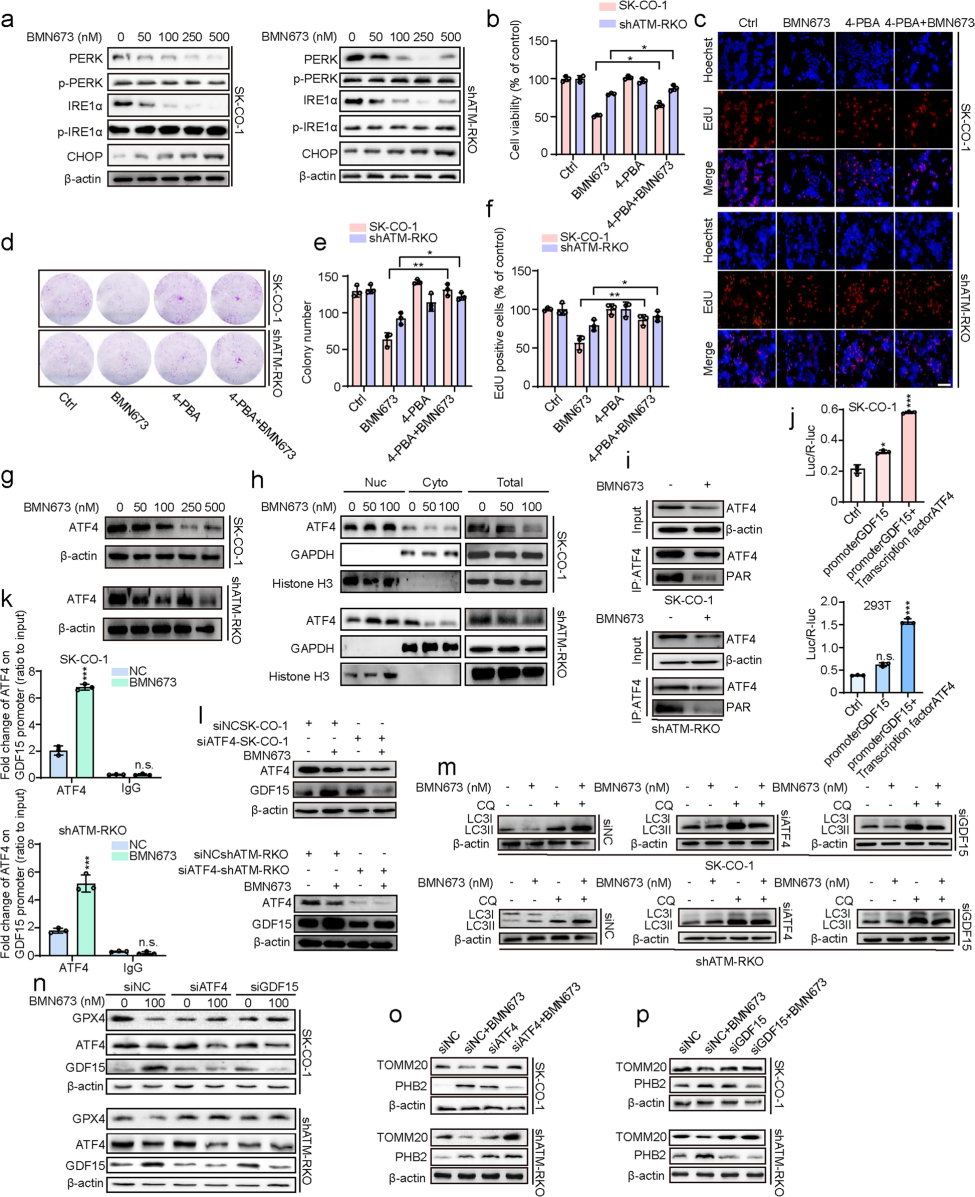
ATF4-GDF15 axis contributes to the anticancer effects of BMN673. **a** Immunoblot analysis of PERK, p-PERK, IRE1α, p-IRE1α and CHOP expression in SK-CO-1 and shATM-RKO cells treated with the indicated concentrations of BMN673 for 48 h. **b** SK-CO-1 and shATM-RKO cells were treated with 50 nM BMN673 in the presence or absence of 2 mM 4-PBA.The Cell viability were examined by CCK8 assay. **c**, **f** SK-CO-1 and shATM-RKO cells treated with vehicle, BMN673 (50 nM), 4-PBA (2 mM), or in combination for 48 h. The EdU incorporation was quantitated. Scale bar, 100 μm. **d**, **e** SK-CO-1 and shATM-RKO cells were treated with 50 nM BMN673 in the presence or absence of 2 mM 4-PBA. Cell proliferation was detected by colony formation assays. **g** Immunoblot analysis of ATF4 expression in SK-CO-1 and shATM-RKO cells treated with the indicated concentrations of BMN673 for 48 h. **h** Immunoblot analysis of ATF4 expression in cytoplasm and nucleus of SK-CO-1 and shATM-RKO cells treated with the indicated concentrations of BMN673 for 48 h. **i** PARylation of ATF4 was assessed by immunoprecipitation with an anti-ATF4 antibody followed by immunoblotting with an anti-poly-ADP-ribose (PAR) antibody in SK-CO-1 and shATM-RKO cells treated with or without 50 nM BMN673 for 48 h. The results demonstrate that ATF4 is covalently modified by PAR. **j** Luciferase reporter assays. SK-CO-1 and 293 T cells were transiently transfected with the indicated plasmids, and 48 h after transfection, the luciferase activities were measured. **k** SK-CO-1 and shATM-RKO cells were treated with DMSO or 50 nM BMN673 for 48 h. Normalized inputs of chromatin DNA from SK-CO-1 and shATM-RKO cells were pulled down with ATF4 or negative IgG antibodies. DNA templates were amplified by PCR. **l** Immunoblot analysis of ATF4 and GDF15 expression in SK-CO-1 and shATM-RKO cells transfected with siNC or siATF4 24 h, followed by treatment with or without 50 nM BMN673 for another 48 h. **m** Immunoblot analysis of LC3B expression in SK-CO-1 and shATM-RKO cells transfected with siNC, siATF4 or siGDF15 for 24 h, followed by treatment with or without 100 nM BMN673 in the presence or absence of 10 μM CQ for another 48 h. **n** Immunoblot analysis of GPX4 expression in SK-CO-1 and shATM-RKO cells transfected with siNC, siATF4 or siGDF15 for 24 h, followed by treatment with or without 100 nM BMN673 for another 48 h. **o**,**p** Immunoblot analysis of TOMM20 and PHB2 expression in SK-CO-1 and shATM-RKO cells transfected with siNC, siATF4 or siGDF15 24 h, followed by treatment with or without 50 nM BMN673 for another 48 h. Data are means ± s.d. and are representative of 3 independent experiments. *, *P* < 0.05, **, *P* < 0.01, ***, *P* < 0.001

The ER stress-mediated PERK-ATF4 signaling pathway emerged as the most significantly altered pathway in the RNA-sequencing analysis, with ER lipid oxidation being a pivotal event in ferroptosis. Thus, the expression levels of ER stress-related signaling pathway proteins were assessed to determine whether BMN673 treatment induced ER stress in ATM-deficient CRC cells. The expression levels of protein kinase R (PKR)-like endoplasmic reticulum kinase (PERK) and Inositol-requiring enzyme 1 alpha (IRE1α) were downregulated compared with the control group, while the levels of phosphorylated PERK (p-PERK) and phosphorylated IRE1α (p-IRE1α) remained largely unaltered. In contrast, the expression of C/EBP homologous protein was notably upregulated (Fig. 5a). 4-Phenylbutyrate (4-PBA), an ER stress inhibitor, was employed to determine the functional involvement of ER stress in the anticancer activity of BMN673. A significant restoration of cell viability was observed in ATM-deficient CRC cells treated concurrently with BMN673 (Fig. 5b). Consistently, a similar increase in cell proliferation was also evidenced using EdU labeling (Fig. 5c, f) and colony formation analysis (Fig. 5d, e). Together, these data underscore the role of ER stress in mediating the anticancer effects of BMN673.

The RNA-sequencing data revealed the paradoxical suppression of ATF4 transcription despite the upregulation of its classical target genes (Fig. 1i, k). ATF4 protein levels were analyzed to elucidate this apparent discrepancy. Although total ATF4 expression decreased upon BMN673 treatment (Fig. 5g), its nuclear accumulation remained unaffected (Fig. 5h). Previous studies have shown that ATF4 undergoes dePARylation under PARP inhibition, enhancing its transcriptional activity. This process mitigates excessive MAPK pathway activation, thereby protecting mitochondria from damage [34]. Co-immunoprecipitation assays were performed to verify this mechanism, and BMN673 treatment was confirmed to significantly decrease the PARylation of ATF4 in ATM-deficient CRC cells (Fig. 5i). These results suggest that BMN673 augments ATF4's transcriptional potential by preventing its PARylation.

ATF4 was knocked down to identify its downstream effectors. The concomitant downregulation of several ATF4 target genes was observed in BMN673-treated ATM-deficient CRC cells, including *GDF15*, *ATF3*, *TRIB3*, *ASNS*, and *SESN2* (Fig. S5a, b). Among these, growth differentiation factor 15 (GDF15) has garnered particular attention due to its pivotal roles in regulating ferroptosis and mitophagy [35–37]. A recent study showed that ATF4 could bind to the GDF15 promoter and positively regulate GDF15 expression in lipopolysaccharide-mediated inflammation [38]. Thus, whether GDF15 serves as a transcriptional target of ATF4 was investigated using dual-luciferase reporter assays, which demonstrated that ATF4 positively regulated GDF15 promoter activity in both SK-CO-1 and 293 T cells (Fig. 5j). Immunohistochemical analysis of tumor tissue from the xenograft model similarly revealed stronger GDF15 staining in the BMN673-treated SK-CO-1 groups compared to the controls (Fig. S5c, d). Whether ATF4 directly regulates GDF15 by binding to its promoter region was examined. Chromatin immunoprecipitation (ChIP) assays revealed the significantly enriched binding of ATF4 to the GDF15 promoter in BMN673-treated ATM-deficient CRC cells (Fig. 5k). Furthermore, ATF4 knockdown abolished the BMN673-induced upregulation of GDF15 (Fig. 5l), confirming GDF15 as a direct transcriptional target of ATF4 in ATM-deficient CRC cells.

CCK8 assays of cells with ATF4 and GDF15 knockdown were performed to investigate the functional significance of the ATF4-GDF15 axis in mediating the anticancer effects of BMN673. ATF4 and GDF15 knockdown significantly attenuated the anticancer effect of BMN673 (Fig. S5e-h). Additionally, the impact of ATF4 and GDF15 knockdown on autophagy, ferroptosis and mitophagy was examined. The results demonstrated that the knockdown of either ATF4 or GDF15 alleviated BMN673-induced LC3-II accumulation (Fig. 5m), restored GPX4 protein levels (Fig. 5n), restored TOMM20 expression (Fig. 5o, p), suppressed PHB2 upregulation (Fig. 5o, p), recovered GSH levels (Fig. S5i, j), and reduced MDA accumulation (Fig. S5k, l). These findings indicate that the ATF4-GDF15 axis coordinately regulates mitophagy, autophagy, and ferroptosis in BMN673-treated ATM-deficient CRC cells.

### BMN673 sensitizes ATM-deficient colorectal cancer cells to RT

**Fig. 6:**
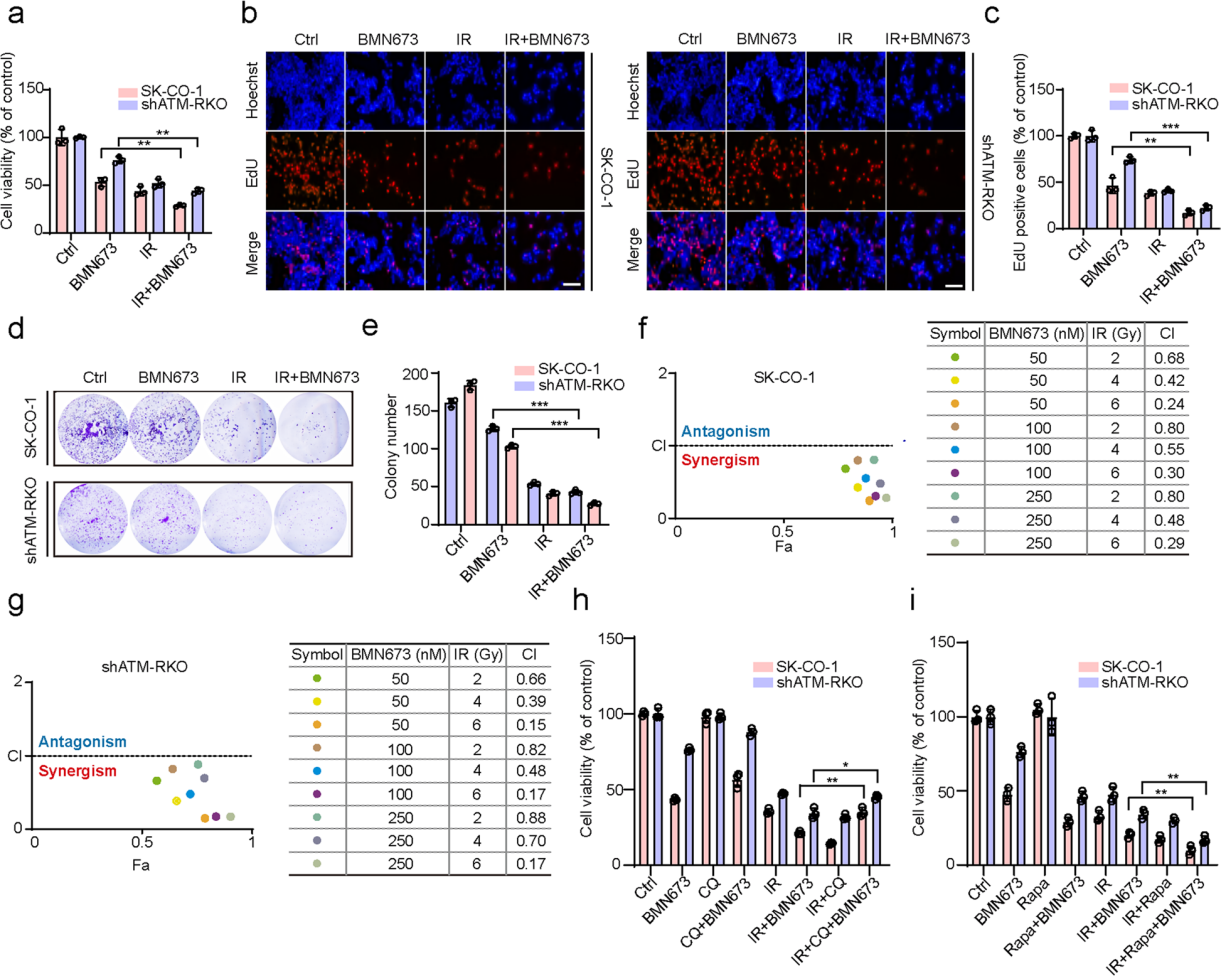
BMN673 sensitizes ATM-deficient colorectal cancer cells to RT. **a** CCK8 assay of SK-CO-1 and shATM-RKO cells treated with 2 Gy IR, followed by treatment with or without 50 nM BMN673 for another 48 h. **b**, **c** SK-CO-1 and shATM-RKO cells treated with 2 Gy IR, followed by treatment with or without 50 nM BMN673 for another 48 h. The EdU incorporation was quantitated. Scale bar, 100 μm. **d**, **e** Colony formation assay of SK-CO-1 and shATM-RKO cells treated with 2 Gy IR, followed by treatment with or without 50 nM BMN673 for another 48 h, cells were then seeded into 12-well plates for two weeks and colony numbers were quantified. **f**, **g** Combination Index (CI) for BMN673 and IR. The CI was calculated by CompuSyn software using SK-CO-1 and shATM-RKO cells. CI > 1, CI = 1, and CI < 1 indicate antagonistic, additive, and synergistic effects, respectively. **h** CCK8 assay of SK-CO-1 and shATM-RKO cells treated with 2 Gy IR, followed by treatment with or without 50 nM BMN673 in the presence or absence of 10 μM CQ for another 48 h. **i** CCK8 assay of SK-CO-1 and shATM-RKO cells treated with 2 Gy IR, followed by treatment with or without 50 nM BMN673 in the presence or absence of 10 μM Rapa for another 48 h. Data are means ± s.d. and are representative of 3 independent experiments. *, *P* < 0.05, **, *P* < 0.01, ***, *P* < 0.001

BMN673 has garnered significant attention as a highly effective radiosensitizer, demonstrating sensitization capabilities superior to other PARP inhibitors [39]. Therefore, whether BMN673 combined with RT had a synergistic anticancer effect on ATM-deficient CRC cells was investigated. Combined treatment with BMN673 and ionizing radiation (IR) significantly decreased the viability of ATM-deficient CRC cells compared to BMN673 or IR alone (Fig. 6a). This finding was further substantiated by decreased EdU incorporation (Fig. 6b, c) and colony formation capacity (Fig. 6d, e). The combined effects of BMN673 and IR were assessed by treating ATM-deficient CRC cells with varying concentrations of BMN673 in combination with different doses of IR. The interaction was analyzed using CompuSyn software to calculate the combination index (CI). The CI values ranged from 0.24 to 0.80 in SK-CO-1 cells and from 0.15 to 0.88 in shATM-RKO cells, indicating moderate to strong synergistic anti-proliferative effects between BMN673 and IR (Fig. 6f, g).

The role of autophagy in mediating this synergistic interaction was investigated by employing CQ, an autophagy inhibitor, in conjunction with IR and BMN673. The CCK8 assay revealed that the combination of IR with CQ enhanced anticancer effects in ATM-deficient CRC cells compared to IR alone, consistent with the results of previous studies suggesting that IR often induces protective autophagy, while autophagy inhibition can amplify radiation sensitivity [40]. However, contrary to expectations, combined treatment with IR and BMN673, accompanied by CQ, restored the viability of ATM-deficient CRC cells (Fig. 6h). Similarly, the colony formation assay yielded results consistent with this finding (Fig. S5m, n). Moreover, the CCK8 assay was performed by employing Rapa, autophagy-inducer, in conjunction with IR and BMN673. The results showed that the addition of Rapa to combined treatment with IR and BMN673 further reduced the viability of ATM-deficient CRC cells (Fig. 6i). These findings suggest that BMN673 might elicit excessive autophagy-associated cell death in these cells, and the inhibition of autophagy by CQ subsequently diminished the overall anticancer effect. These findings underscore the complex interplay between autophagy and RT, wherein moderate levels of autophagy can provide protective benefits against radiation-induced stress, whereas excessive autophagy might amplify radiosensitivity.

## Discussion

The impairment of the DNA damage repair (DDR) pathway presents a novel therapeutic avenue for targeted cancer treatment. Recent investigations have underscored the emerging significance of germline pathogenic variants in HRR-related genes, notably *BRCA1*, *ATM*, and *PALB2*, as predisposing factors for CRC, particularly early-onset cases [41–43]. Notably, ATM, a DDR kinase, is frequently mutated in a wide spectrum of solid and hematological tumors, and its involvement has been documented in up to 9–15% of CRC [44–48]. PARP inhibitors, particularly BMN673, have emerged as potent therapeutic agents, exerting synthetic lethality in cancers deficient in HRR [49, 50]. This study demonstrated that BMN673 could significantly inhibit growth of ATM-deficient colorectal cancer cells through synthetic lethal. BMN673 could also trigger autophagy-associated cell death, ferroptosis, mitochondrial dysfunction, and mitophagy, collectively contributing to its anticancer effects in ATM-deficient CRC cells. Moreover, multiple studies have indicated that BMN673 enhances radiation sensitivity more effectively than other PARP inhibitors [51]. This study also found that BMN673 significantly enhanced the sensitivity of ATM-deficient CRC cells to RT, and excessive autophagy, induced by BMN673, exacerbated RT-mediated cell death.

Autophagy plays dual roles in tumor progression. Previous studies have shown that PARP inhibitors promote autophagic flux in various cancers [52–54]. This study first discovered that BMN673 induced autophagy-associated cell death in ATM-deficient CRC cells. Although RT routinely triggers protective autophagy [55], some studies have proposed that autophagic cell death can sensitize tumors to irradiation [56, 57]. The findings of the current study consistently showed that BMN673-induced autophagy-associated cell death enhanced the sensitivity of ATM-deficient CRC cells to RT, distinct from radiation-induced protective autophagy. This discrepancy underscores the complex interplay between autophagy and RT, which can be strategically modulated to improve therapeutic outcomes in ATM-deficient CRC patients.

Ferroptosis, a novel iron-dependent form of regulated cell death, stands apart from apoptosis, necrosis, and autophagy in its distinct mechanisms and morphological features [20]. The core regulatory system comprising SLC7A11-mediated cystine transport and subsequent GSH synthesis, along with GPX4, governs ferroptosis [54, 58]. Although DNA damage is not the primary mediator of ferroptosis, studies have increasingly demonstrated that PARP inhibitors can promote ferroptosis [59]. The present study findings underscore BMN673’s ability to induce ferroptosis in ATM-deficient CRC cells by downregulating GPX4 expression and augmenting lipid peroxidation, independently of SLC7A11 expression. This pattern aligns with other reported mechanisms of GPX4-targeted ferroptosis induction [60, 61]. The ability of BMN673 to potentiate ferroptosis may underlie its superior radiosensitization properties compared to other PARP inhibitors [39].

Aberrant mitochondrial metabolism is a hallmark of cancer cells, rendering them vulnerable targets for therapeutic intervention [62, 63]. Notably, during ferroptosis, mitochondria typically exhibit atrophy and a dense state [64]. The present study results demonstrated that BMN673 induced morphological shrinkage, mass reduction, and decreased membrane potential in mitochondria in ATM-deficient CRC cells, indicating impaired mitochondrial function. Intriguingly, reduced ROS accumulation and the upregulation of mitochondrial function-related metabolites may be attributed to enhanced mitophagy. Several studies showed that DNA damage could indirectly trigger mitophagy [65]. The present findings elucidated a dual-pronged mechanism for mitophagy induction by BMN673; besides LC3-mediated conventional mitophagy, BMN673 induced mitophagy through SPATA18, a p53-inducible protein crucial for mitochondrial quality control [66]. This dual induction of mitophagy highlights the intricate balance between mitochondrial damage and the compensatory responses of tumor cells.

ATF4, a pivotal transcription factor, orchestrates the expression of genes involved in protein folding, amino acid metabolism, autophagy, and redox balance [67–69]. PARP inhibition stimulates MAPK phosphatase 1 (MKP-1) expression through ATF4 dePARylation, thereby diminishing ROS-induced cell death, decreasing mitochondrial ROS generation, and preserving mitochondrial membrane potential [70]. The present study revealed that although ATF4 expression was suppressed by BMN673, its PARPylation was inhibited, leading to ATF4 activation and the subsequent upregulation of GDF15. GDF15, a member of the transforming growth factor (TGF)-β superfamily, plays a critical role in regulating mitochondrial function, ferroptosis, and autophagy [71–73]. It has been implicated in hepatocellular carcinoma progression in an autophagy-dependent manner [74] and serves as a ferroptosis gene signature predicting CRC prognosis [75]. The present study findings indicate that BMN673-induced ferroptosis, autophagy-associated cell death, and mitophagy in ATM-deficient CRC cells are dependent on the ATF4-GDF15 axis. The selective activation of this pathway may stem from synergistic effects between ATM deficiency and PARP inhibition. ATM deficiency leads to elevated replication stress and genomic instability [76, 77], and BMN673-induced DNA damage synergizes with these pre-existing vulnerabilities, creating a dual-hit effect that exacerbates ER stress and specifically activates the integrated stress response pathway [78]. As a central regulator of cellular homeostasis, severe ATM deficiency likely amplifies and accelerates the cellular stress response [79, 80]. The present data indicate that this synergistic stress specifically affects ATF4, the core effector of the integrated stress response.

However, a key limitation of this study is that the specific downstream effectors of GDF15 in regulating ferroptosis, autophagy-associated cell death, and mitophagy remain incompletely elucidated, and the temporal hierarchy between these pathways is unclear. Furthermore, this research relied on cell lines and mouse models without validation in primary patient-derived samples. These limitations highlight important directions for future research, which include identifying GDF15 downstream targets and tracking ATF4 and GDF15 expression levels in cellular models and patient specimens.

**Fig. 7:**
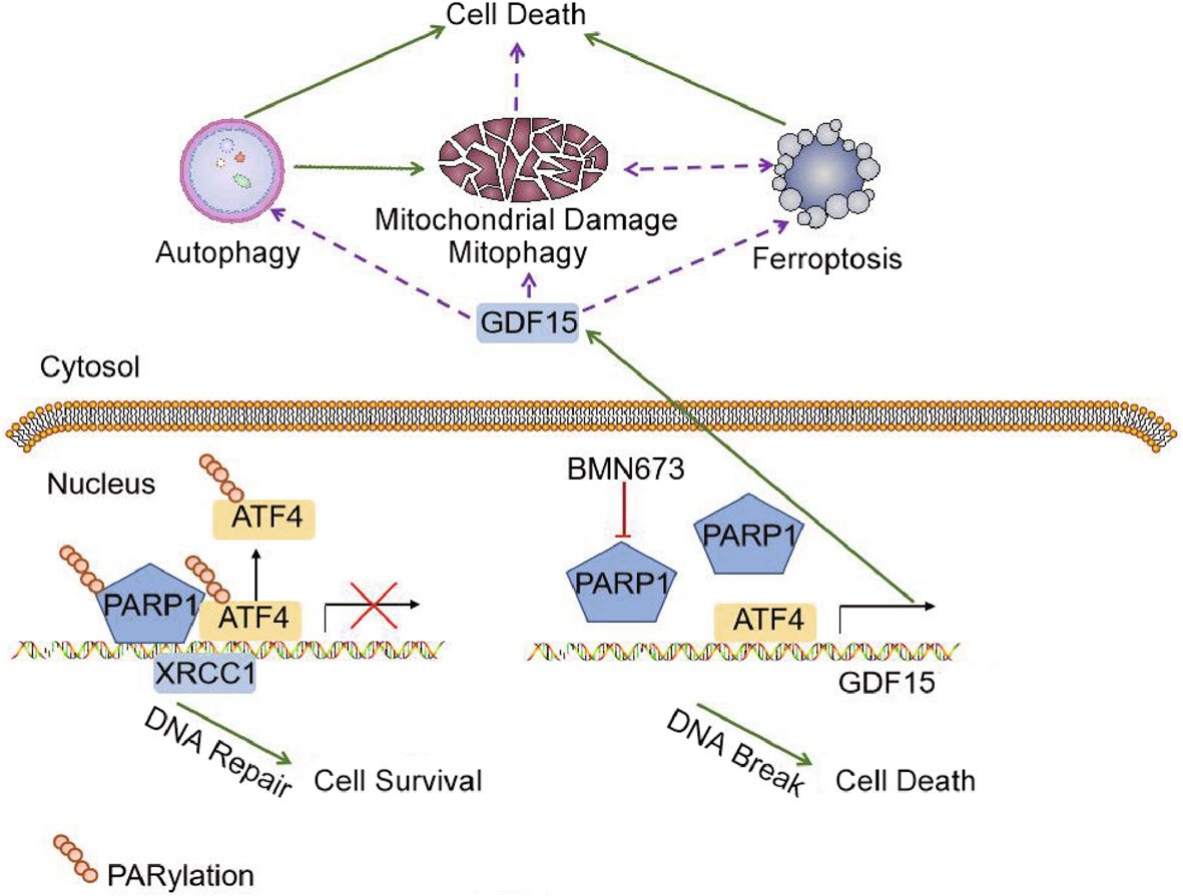
Proposing model of the mechanisms of BMN673-mediated anticancer effect. BMN673 promotes cell death by synthetic lethality and induces GDF15 expression by inhibiting the poly adenosine diphosphate ribosylation modification of ATF4 to attract ATF4 to the GDF15 promoter region. GDF15 overexpression regulates the sensitivity of ATM-deficient colorectal cancer cells to BMN673 by promoting autophagy-associated cell death, ferroptosis and mitophagy

Taken together, the results identified a novel PARylation-regulated ATF4-GDF15 pathway governing autophagy, ferroptosis, and mitophagy in ATM-deficient CRC cells (Fig. 7), which can contribute to BMN673-induced anticancer effects alone or in combination with RT. The findings redefine the therapeutic paradigm beyond DNA damage repair, positioning the ATF4-GDF15 axis as a pivotal biomarker and promising therapeutic target for ATM-deficient cancers. Future work will focus on delineating the precise downstream effectors of GDF15 and validating this axis in clinical cohorts.

## Materials and methods

### Reagents and antibodies

BMN673 (S7048) was purchased from Selleck. Chloroquine (HY-17589A), bafilomycin A1 (HY-100558), rapamycin (HY-10219), and KU-55933 (HY-12016) were purchased from MedChemExpress. 4-PBA (SML0309) was purchased from Sigma. Antibodies are shown in Supplementary Table 3.

### Cell culture

Human CRC cell lines SW480, RKO, HCT116, and SW620 were obtained from the cell bank of the Chinese Academy of Science, and SK-CO-1 was obtained from Cobioer. Cells were maintained in high-glucose Dulbecco’s Modified Eagle Medium (DMEM, Gibco), Roswell Park Memorial Institute 1640 (RPMI 1640, Gibco) and Minimum Essential Medium (MEM, Gibco) supplemented with 10% fetal bovine serum (FBS, ExCell Bio), penicillin (100 U/mL) and streptomycin (100 U/mL). All cells were grown at 37℃ in a humidified incubator with 5% CO_2_. Cell lines were tested by short-tandem repeat analysis, and the last time of authentication was June 2023. All cell lines used in this study were regularly tested by mycoplasma PCR detection and confirmed to be free of mycoplasma contamination.

### Animal study

All animal experiments were approved by the Laboratory Animal Management and Use Committee of Chongqing Medical University. All procedures used adhered to the regulations of the Laboratory Animal Management and Use Committee of Chongqing Medical University (IACUC-CQMU-2023–12089). A total of eight female Balb/c nude mice (4 weeks old) were obtained from Huafukang Bioscience Co., Ltd (Beijing, China). All mice were housed in cages containing Aspen chip bedding in rooms under standard conditions (temperature: 20–22 °C, relative humidity: 61–65%, 12 h light/dark cycle, five mice per cage) in accordance with institutional guidelines from the Laboratory Animal Management and Use Committee of Chongqing Medical University. The mice had free access to tap water and standard commercial mouse chow.

For the subcutaneous xenograft tumor models with human cell lines SK-CO-1, exponentially growing SK-CO-1 cells (8 × 10^6^ cells per 100 µL of diluted RPMI 1640 (1:1 dilution in Matrigel)) were injected into the left flank of the Balb/c nude mice. Treatment began 15 days post-tumor implantation after palpable tumor formation. The mice were randomized into different groups prior to treatment (*n* = 4 for each group). According to the treatments, mice were grouped into: (1) vehicle alone (100 μL, 0.5% dimethyl sulfoxide (DMSO) in PBS) and (2) BMN673 (2 mg/kg/day; 100 μL; 0.5% DMSO in PBS) groups. Treatment of the vehicle and BMN673 groups was administered through oral gavage (per os) on alternate days over a 15-day period. Tumor volume was calculated using digital caliper measurements. The mice were euthanized for analysis on day 30. Tumors were harvested and frozen in liquid nitrogen or fixed in 4% formalin immediately.

### Luciferase reporter constructs

The promoter regions of *GDF15* (−1232/+ 488) were obtained by PCR-based amplification and cloned into a pGL4.1-Basic vector to generate reporters. Cells were seeded into 12-well plates in triplicate and co-transfected with the corresponding plasmids. Then, luciferase activities were measured using the dual-luciferase assay system (Promega).

### Mitophagy detection

Mitophagy in SK-CO-1 and shATM-RKO cells was detected using the Mitophagy Detection Kit (Dojindo, MD01). Briefly, cells were seeded in 35 mm glass-bottom dishes overnight and subjected to different treatments. The cells were then incubated with Mitophagy Dye (100 nM) for 30 min. After washing with PBS, fluorescence images were captured using a confocal microscope (Leica).

### Chromatin immunoprecipitation

ChIP was performed using an EZ-Magna Chromatin Immunoprecipitation A/G kit (Millipore) according to the manufacturer’s instructions. Briefly, SK-CO-1 and shATM-RKO cells were cultured with or without 100 nM BMN673 for 48 h. Chromatin solutions were sonicated, incubated with an anti-ATF4 antibody or control IgG, and rotated overnight at 4 °C. Then, the enriched chromatin DNA was purified and subjected to PCR analysis. The following primer sets were used to amplify the human GDF15 promoter region containing the ATF4-binding site: GDF15 ChIP forward, 5’ -CCGAAGACTCCAGATTCCGA-3’ and GDF15 ChIP reverse, 5’ -CCCGAGAGATACGCAGGTG-3’.

### Data analysis and statistics

All statistical analyses were performed using SPSS 16.0 software (SPSS Inc., Chicago, IL, USA). Qualitative variables were compared using Chi-square tests, whereas quantitative variables were analyzed using the Student’s t-test. Data are expressed as mean ± SD. The difference between different groups was evaluated using Student’s t-tests or Mann–Whitney U tests. Data were analyzed using GraphPad Prism software version 8.30 (GraphPad software, San Diego, CA, USA). A P-value of < 0.05 was considered statistically significant.

## Supplementary Information

Supplementary Material 1.

## Data Availability

All data needed to evaluate the conclusions are included in this article and/or in its supplemental material. The data within the study are available upon request from the corresponding author.

## References

[1] Siegel R, Desantis C, Jemal A. Colorectal cancer statistics, 2014. CA Cancer J Clin. 2014;64(2):104–17. 10.3322/caac.21220. 24639052 10.3322/caac.21220

[2] Siegel RL, Miller KD, Fuchs HE, Jemal A. Cancer statistics, 2021. CA Cancer J Clin. 2021;71(1):7–33. 10.3322/caac.21654. 33433946 10.3322/caac.21654

[3] Matsuda T, Fujimoto A, Igarashi Y. Colorectal cancer: epidemiology, risk factors, and public health strategies. Digestion. 2025;106(2):91–9. 10.1159/000543921. 39938491 10.1159/000543921

[4] Siegel RL, Giaquinto AN, Jemal A. Cancer statistics, 2024. CA Cancer J Clin. 2024;74(1):12–49. 10.3322/caac.21820. 38230766 10.3322/caac.21820

[5] Chen W, Zheng R, Zhang S, Zeng H, Zuo T, Xia C, et al. Cancer incidence and mortality in China in 2013: an analysis based on urbanization level. Chin J Cancer Res. 2017;29(1):1–10. 10.21147/j.issn.1000-9604.2017.01.01. 28373748 10.21147/j.issn.1000-9604.2017.01.01 PMC5348470

[6] Chen W, Zheng R, Baade PD, Zhang S, Zeng H, Bray F, et al. Cancer statistics in China, 2015. CA Cancer J Clin. 2016;66(2):115–32. 10.3322/caac.21338. 26808342 10.3322/caac.21338

[7] Feng YJ, Wang N, Fang LW, Cong S, Yin P, Li YC, et al. Burden of disease of colorectal cancer in the Chinese population, in 1990 and 2013. Zhonghua Liu Xing Bing Xue Za Zhi. 2016;37(6):768–72. 10.3760/cma.j.issn.0254-6450.2016.06.005. 27346099 10.3760/cma.j.issn.0254-6450.2016.06.005

[8] Flippot R, Patrikidou A, Aldea M, Colomba E, Lavaud P, Albigès L, et al. PARP inhibition, a new therapeutic avenue in patients with prostate cancer. Drugs. 2022;82(7):719–33. 10.1007/s40265-022-01703-5. 35511402 10.1007/s40265-022-01703-5

[9] Ge M, Luo J, Wu Y, Shen G, Kuang XJMO. The biological essence of synthetic lethality: bringing new opportunities for cancer therapy. MedComm – Oncology. 2024;3(1):e70.

[10] Mateo J, Lord CJ, Serra V, Tutt A, Balmaña J, Castroviejo-Bermejo M, et al. A decade of clinical development of PARP inhibitors in perspective. Ann Oncol. 2019;30(9):1437–47. 10.1093/annonc/mdz192. 31218365 10.1093/annonc/mdz192 PMC6771225

[11] Pasupulati SL, Srinivasa Rao K, Sharma S, Madhavrao C, Rangari G, Misra AK, et al. Clinical pharmacology and therapeutic applications of talazoparib: a comprehensive review. Cancer Chemother Pharmacol. 2025;95(1):87. 10.1007/s00280-025-04810-8. 40956449 10.1007/s00280-025-04810-8

[12] Pellegrino B, Mateo J, Serra V, Balmaña J. Controversies in oncology: are genomic tests quantifying homologous recombination repair deficiency (HRD) useful for treatment decision making? ESMO Open. 2019;4(2):e000480. 10.1136/esmoopen-2018-000480. 31231558 10.1136/esmoopen-2018-000480 PMC6555601

[13] Gewirtz DA. The four faces of autophagy: implications for cancer therapy. Cancer Res. 2014;74(3):647–51. 10.1158/0008-5472.Can-13-2966. 24459182 10.1158/0008-5472.CAN-13-2966

[14] Arun B, Akar U, Gutierrez-Barrera AM, Hortobagyi GN, Ozpolat B. The PARP inhibitor AZD2281 (olaparib) induces autophagy/mitophagy in BRCA1 and BRCA2 mutant breast cancer cells. Int J Oncol. 2015;47(1):262–8. 10.3892/ijo.2015.3003. 25975349 10.3892/ijo.2015.3003 PMC6904111

[15] Liu Y, Song H, Song H, Feng X, Zhou C, Huo Z. Targeting autophagy potentiates the anti-tumor effect of PARP inhibitor in pediatric chronic myeloid leukemia. AMB Express. 2019;9(1):108. 10.1186/s13568-019-0836-z. 31309361 10.1186/s13568-019-0836-z PMC6629728

[16] Santiago-O’Farrill JM, Weroha SJ, Hou X, Oberg AL, Heinzen EP, Maurer MJ, et al. Poly(adenosine diphosphate ribose) polymerase inhibitors induce autophagy-mediated drug resistance in ovarian cancer cells, xenografts, and patient-derived xenograft models. Cancer. 2020;126(4):894–907. 10.1002/cncr.32600. 31714594 10.1002/cncr.32600 PMC6992526

[17] Zai W, Chen W, Han Y, Wu Z, Fan J, Zhang X, et al. Targeting PARP and autophagy evoked synergistic lethality in hepatocellular carcinoma. Carcinogenesis. 2020;41(3):345–57. 10.1093/carcin/bgz104. 31175354 10.1093/carcin/bgz104

[18] Lesage J, DiMauro A, Schab AM, Stidham S, Mullen MM, Fuh KC, et al. DDR2 confers ferroptosis resistance to cancer-associated fibroblasts and attenuates PARPi sensitivity of ovarian tumor cells. Mol Cancer Res. 2025. 10.1158/1541-7786.Mcr-25-0268. 10.1158/1541-7786.MCR-25-0268 PMC12700032 40906573

[19] Zhang J, Ouyang D, Liu M, Xiang Y, Li Z. Research progress on ferroptosis and PARP inhibitors in ovarian cancer: action mechanisms and resistance mechanisms. Front Pharmacol. 2025;16:1598279. 10.3389/fphar.2025.1598279. 40342999 10.3389/fphar.2025.1598279 PMC12058875

[20] Bebber CM, Müller F, Prieto Clemente L, Weber J, von Karstedt S. Ferroptosis in cancer cell biology. Cancers (Basel). 2020;12(1):164. 10.3390/cancers12010164. 31936571 10.3390/cancers12010164 PMC7016816

[21] Guo K, Lu M, Bi J, Yao T, Gao J, Ren F, et al. Ferroptosis: mechanism, immunotherapy and role in ovarian cancer. Front Immunol. 2024;15:1410018. 10.3389/fimmu.2024.1410018. 39192972 10.3389/fimmu.2024.1410018 PMC11347334

[22] Xie X, Chen C, Wang C, Guo Y, Sun B, Tian J, et al. Targeting GPX4-mediated ferroptosis protection sensitizes BRCA1-deficient cancer cells to PARP inhibitors. Redox Biol. 2024;76:103350. 10.1016/j.redox.2024.103350. 39265497 10.1016/j.redox.2024.103350 PMC11415882

[23] Hetz C, Papa FR. The unfolded protein response and cell fate control. Mol Cell. 2018;69(2):169–81. 10.1016/j.molcel.2017.06.017. 29107536 10.1016/j.molcel.2017.06.017

[24] Ye J, Koumenis C. ATF4, an ER stress and hypoxia-inducible transcription factor and its potential role in hypoxia tolerance and tumorigenesis. Curr Mol Med. 2009;9(4):411–6. 10.2174/156652409788167096. 19519398 10.2174/156652409788167096

[25] Deng K, Li Q, Lu L, Wang L, Cheng Z, Wang S. Proteasome and PARP1 dual-target inhibitor for multiple myeloma: fluzoparib. Biochem Biophys Rep. 2024;39:101781. 10.1016/j.bbrep.2024.101781. 39071914 10.1016/j.bbrep.2024.101781 PMC11279668

[26] Majera D, Skrott Z, Bouchal J, Bartkova J, Simkova D, Gachechiladze M, et al. Targeting genotoxic and proteotoxic stress-response pathways in human prostate cancer by clinically available PARP inhibitors, vorinostat and disulfiram. Prostate. 2019;79(4):352–62. 10.1002/pros.23741. 30499118 10.1002/pros.23741

[27] Fan H, Xu Z, Yao K, Zheng B, Zhang Y, Wang X, et al. Osteoclast cancer cell metabolic cross-talk confers PARP inhibitor resistance in bone metastatic breast cancer. Cancer Res. 2024;84(3):449–67. 10.1158/0008-5472.Can-23-1443. 38038966 10.1158/0008-5472.CAN-23-1443

[28] Maillet P, Chappuis PO, Vaudan G, Dobbie Z, Müller H, Hutter P, et al. A polymorphism in the ATM gene modulates the penetrance of hereditary non-polyposis colorectal cancer. Int J Cancer. 2000;88(6):928–31. 10.1002/1097-0215(20001215)88:6%3c928::aid-ijc14%3e3.0.co;2-p. 11093816 10.1002/1097-0215(20001215)88:6<928::aid-ijc14>3.0.co;2-p

[29] Sriramulu S, Ramachandran M, Subramanian S, Kannan R, Gopinath M, Sollano J, et al. A review on role of ATM gene in hereditary transfer of colorectal cancer. Acta Biomed. 2019;89(4):463–469. 10.23750/abm.v89i4.6095. 10.23750/abm.v89i4.6095 PMC6502098 30657113

[30] Zhao C, Yu D, He Z, Bao L, Feng L, Chen L, et al. Endoplasmic reticulum stress-mediated autophagy activation is involved in cadmium-induced ferroptosis of renal tubular epithelial cells. Free Radic Biol Med. 2021;175:236–48. 10.1016/j.freeradbiomed.2021.09.008. 34520822 10.1016/j.freeradbiomed.2021.09.008

[31] Santini SJ, Cordone V, Falone S, Mijit M, Tatone C, Amicarelli F, et al. Role of mitochondria in the oxidative stress induced by electromagnetic fields: focus on reproductive systems. Oxid Med Cell Longev. 2018;2018:5076271. 10.1155/2018/5076271. 30533171 10.1155/2018/5076271 PMC6250044

[32] Youle RJ, Narendra DP. Mechanisms of mitophagy. Nat Rev Mol Cell Biol. 2011;12(1):9–14. 10.1038/nrm3028. 21179058 10.1038/nrm3028 PMC4780047

[33] Dan X, Babbar M, Moore A, Wechter N, Tian J, Mohanty JG, et al. DNA damage invokes mitophagy through a pathway involving Spata18. Nucleic Acids Res. 2020;48(12):6611–23. 10.1093/nar/gkaa393. 32453416 10.1093/nar/gkaa393 PMC7337932

[34] Gallyas F, Jr., Sumegi B. Mitochondrial Protection by PARP Inhibition. Int J Mol Sci. 2020;21(8). 10.3390/ijms21082767. 10.3390/ijms21082767 PMC7215481 32316192

[35] Chen L, Qiao L, Bian Y, Sun X. GDF15 knockdown promotes erastin-induced ferroptosis by decreasing SLC7A11 expression. Biochem Biophys Res Commun. 2020;526(2):293–9. 10.1016/j.bbrc.2020.03.079. 32209255 10.1016/j.bbrc.2020.03.079

[36] Xia M, Zhang Q, Zhang Y, Li R, Zhao T, Chen L, et al. Growth differentiation factor 15 regulates oxidative stress-dependent ferroptosis post spinal cord injury by stabilizing the p62-Keap1-Nrf2 signaling pathway. Front Aging Neurosci. 2022;14:905115. 10.3389/fnagi.2022.905115. 35860670 10.3389/fnagi.2022.905115 PMC9289442

[37] Chen RY, Li DW, Xie H, Liu XW, Zhuang SY, Wu HY, et al. Gene signature and prediction model of the mitophagy-associated immune microenvironment in renal ischemia-reperfusion injury. Front Immunol. 2023;14:1117297. 10.3389/fimmu.2023.1117297. 37056767 10.3389/fimmu.2023.1117297 PMC10086170

[38] Li A, Zhao F, Zhao Y, Liu H, Wang Z. ATF4-mediated GDF15 suppresses LPS-induced inflammation and MUC5AC in human nasal epithelial cells through the PI3K/Akt pathway. Life Sci. 2021;275:119356. 10.1016/j.lfs.2021.119356. 33737080 10.1016/j.lfs.2021.119356

[39] Soni A, Lin X, Mladenov E, Mladenova V, Stuschke M, Iliakis G. BMN673 Is a PARP Inhibitor with Unique Radiosensitizing Properties: Mechanisms and Potential in Radiation Therapy. Cancers (Basel). 2022;14(22). 10.3390/cancers14225619. 10.3390/cancers14225619 PMC9688666 36428712

[40] Xin Y, Jiang F, Yang C, Yan Q, Guo W, Huang Q, et al. Role of autophagy in regulating the radiosensitivity of tumor cells. J Cancer Res Clin Oncol. 2017;143(11):2147–57. 10.1007/s00432-017-2487-2. 28786037 10.1007/s00432-017-2487-2 PMC11819394

[41] Mukhopadhyay A, Elattar A, Cerbinskaite A, Wilkinson SJ, Drew Y, Kyle S, et al. Development of a functional assay for homologous recombination status in primary cultures of epithelial ovarian tumor and correlation with sensitivity to poly(ADP-ribose) polymerase inhibitors. Clin Cancer Res. 2010;16(8):2344–51. 10.1158/1078-0432.Ccr-09-2758. 20371688 10.1158/1078-0432.CCR-09-2758

[42] Konstantinopoulos PA, Spentzos D, Karlan BY, Taniguchi T, Fountzilas E, Francoeur N, et al. Gene expression profile of BRCAness that correlates with responsiveness to chemotherapy and with outcome in patients with epithelial ovarian cancer. J Clin Oncol. 2010;28(22):3555–61. 10.1200/jco.2009.27.5719. 20547991 10.1200/JCO.2009.27.5719 PMC2917311

[43] Jenner ZB, Sood AK, Coleman RL. Evaluation of rucaparib and companion diagnostics in the PARP inhibitor landscape for recurrent ovarian cancer therapy. Future Oncol. 2016;12(12):1439–56. 10.2217/fon-2016-0002. 27087632 10.2217/fon-2016-0002 PMC4976841

[44] Oh M, McBride A, Yun S, Bhattacharjee S, Slack M, Martin JR, et al. *BRCA1* and *BRCA2* gene mutations and colorectal cancer risk: systematic review and meta-analysis. J Natl Cancer Inst. 2018;110(11):1178–89. 10.1093/jnci/djy148. 30380096 10.1093/jnci/djy148

[45] Mauri G, Arena S, Siena S, Bardelli A, Sartore-Bianchi A. The DNA damage response pathway as a land of therapeutic opportunities for colorectal cancer. Ann Oncol. 2020;31(9):1135–47. 10.1016/j.annonc.2020.05.027. 32512040 10.1016/j.annonc.2020.05.027

[46] Knijnenburg TA, Wang L, Zimmermann MT, Chambwe N, Gao GF, Cherniack AD, et al. Genomic and Molecular Landscape of DNA Damage Repair Deficiency across The Cancer Genome Atlas. Cell Rep. 2018;23(1):239-254.e236. 10.1016/j.celrep.2018.03.076. 29617664 10.1016/j.celrep.2018.03.076 PMC5961503

[47] Heeke AL, Pishvaian MJ, Lynce F, Xiu J, Brody JR, Chen WJ, et al. Prevalence of Homologous Recombination-Related Gene Mutations Across Multiple Cancer Types. JCO Precis Oncol. 2018;2018. 10.1200/po.17.00286. 10.1200/PO.17.00286 PMC6139373 30234181

[48] Moretto R, Elliott A, Zhang J, Arai H, Germani MM, Conca V, et al. Homologous recombination deficiency alterations in colorectal cancer: clinical, molecular, and prognostic implications. J Natl Cancer Inst. 2022;114(2):271–9. 10.1093/jnci/djab169. 34469533 10.1093/jnci/djab169 PMC8826505

[49] Ellisen LW. PARP inhibitors in cancer therapy: promise, progress, and puzzles. Cancer Cell. 2011;19(2):165–7. 10.1016/j.ccr.2011.01.047. 21316599 10.1016/j.ccr.2011.01.047 PMC3070420

[50] Castro E, Mateo J, Olmos D, de Bono JS. Targeting DNA repair: the role of PARP inhibition in the treatment of castration-resistant prostate cancer. Cancer J. 2016;22(5):353–6. 10.1097/ppo.0000000000000219. 27749330 10.1097/PPO.0000000000000219

[51] Lin X, Soni A, Hessenow R, Sun Y, Mladenov E, Guberina M, et al. Talazoparib enhances resection at DSBs and renders HR-proficient cancer cells susceptible to Polθ inhibition. Radiother Oncol. 2024;200:110475. 10.1016/j.radonc.2024.110475. 39147034 10.1016/j.radonc.2024.110475

[52] Pai Bellare G, Saha B, Patro BS. Targeting autophagy reverses de novo resistance in homologous recombination repair proficient breast cancers to PARP inhibition. Br J Cancer. 2021;124(7):1260–74. 10.1038/s41416-020-01238-0. 33473172 10.1038/s41416-020-01238-0 PMC8007595

[53] Santiago-O’Farrill JM, Blessing Bollu A, Yang H, Orellana V, Pina M, Zhang X, et al. Crizotinib Enhances PARP Inhibitor Efficacy in Ovarian Cancer Cells and Xenograft Models by Inducing Autophagy. Mol Cancer Res. 2024;22(9):840–51. 10.1158/1541-7786.Mcr-23-0680. 38780897 10.1158/1541-7786.MCR-23-0680 PMC11372360

[54] Zhang Z, Lian X, Xie W, Quan J, Liao M, Wu Y, et al. Role of PARP1-mediated autophagy in EGFR-TKI resistance in non-small cell lung cancer. Sci Rep. 2020;10(1):20924. 10.1038/s41598-020-77908-z. 33262410 10.1038/s41598-020-77908-z PMC7708842

[55] Wang Y, Yin W, Zhu X. Blocked autophagy enhances radiosensitivity of nasopharyngeal carcinoma cell line CNE-2 *in vitro*. Acta Otolaryngol. 2014;134(1):105–10. 10.3109/00016489.2013.844365. 24256039 10.3109/00016489.2013.844365

[56] Chang L, Graham PH, Hao J, Ni J, Bucci J, Cozzi PJ, et al. PI3K/Akt/mTOR pathway inhibitors enhance radiosensitivity in radioresistant prostate cancer cells through inducing apoptosis, reducing autophagy, suppressing NHEJ and HR repair pathways. Cell Death Dis. 2014;5(10):e1437. 10.1038/cddis.2014.415. 25275598 10.1038/cddis.2014.415 PMC4237243

[57] Li Q, Xia L, Sun C, Zhang H, Zheng M, Zhang H, et al. Role of borneol induced autophagy in enhancing radiosensitivity of malignant glioma. Front Oncol. 2021;11:749987. 10.3389/fonc.2021.749987. 34917504 10.3389/fonc.2021.749987 PMC8668811

[58] Sun L, Dong H, Zhang W, Wang N, Ni N, Bai X, et al. Lipid peroxidation, GSH depletion, and SLC7A11 inhibition are common causes of EMT and ferroptosis in A549 cells, but different in specific mechanisms. DNA Cell Biol. 2021;40(2):172–83. 10.1089/dna.2020.5730. 33351681 10.1089/dna.2020.5730

[59] Shen D, Luo J, Chen L, Ma W, Mao X, Zhang Y, et al. Parpi treatment enhances radiotherapy-induced ferroptosis and antitumor immune responses via the cGAS signaling pathway in colorectal cancer. Cancer Lett. 2022;550:215919. 10.1016/j.canlet.2022.215919. 36116741 10.1016/j.canlet.2022.215919

[60] Huang B, Wang H, Liu S, Hao M, Luo D, Zhou Y, et al. Palmitoylation-dependent regulation of GPX4 suppresses ferroptosis. Nat Commun. 2025;16(1):867. 10.1038/s41467-025-56344-5. 39833225 10.1038/s41467-025-56344-5 PMC11746948

[61] Zhang Z, Hu Q, Ye S, Xiang L. Inhibition of the PIN1-NRF2/GPX4 axis imparts sensitivity to cisplatin in cervical cancer cells. Acta Biochim Biophys Sin (Shanghai). 2022;54(9):1325–35. 10.3724/abbs.2022109. 35983979 10.3724/abbs.2022109 PMC9827814

[62] Grasso D, Zampieri LX, Capelôa T, Van de Velde JA, Sonveaux P. Mitochondria in cancer. Cell Stress. 2020;4(6):114–46. 10.15698/cst2020.06.221. 32548570 10.15698/cst2020.06.221 PMC7278520

[63] Liu Y, Shi Y. Mitochondria as a target in cancer treatment. MedComm. 2020;1(2):129–39. 10.1002/mco2.16. 34766113 10.1002/mco2.16 PMC8491233

[64] Liu Y, Lu S, Wu LL, Yang L, Yang L, Wang J. The diversified role of mitochondria in ferroptosis in cancer. Cell Death Dis. 2023;14(8):519. 10.1038/s41419-023-06045-y. 37580393 10.1038/s41419-023-06045-y PMC10425449

[65] Babbar M, Basu S, Yang B, Croteau DL, Bohr VA. Mitophagy and DNA damage signaling in human aging. Mech Ageing Dev. 2020;186:111207. 10.1016/j.mad.2020.111207. 31923475 10.1016/j.mad.2020.111207 PMC7047626

[66] Sugimura-Nagata A, Koshino A, Nagao K, Nagano A, Komura M, Ueki A, et al. SPATA18 Expression Predicts Favorable Clinical Outcome in Colorectal Cancer. Int J Mol Sci. 2022;23(5). 10.3390/ijms23052753. 10.3390/ijms23052753 PMC8910917 35269894

[67] Deegan S, Saveljeva S, Gorman AM, Samali A. Stress-induced self-cannibalism: on the regulation of autophagy by endoplasmic reticulum stress. Cell Mol Life Sci. 2013;70(14):2425–41. 10.1007/s00018-012-1173-4. 23052213 10.1007/s00018-012-1173-4 PMC11113399

[68] Harding HP, Zhang Y, Bertolotti A, Zeng H, Ron D. Perk is essential for translational regulation and cell survival during the unfolded protein response. Mol Cell. 2000;5(5):897–904. 10.1016/s1097-2765(00)80330-5. 10882126 10.1016/s1097-2765(00)80330-5

[69] Harding HP, Zhang Y, Zeng H, Novoa I, Lu PD, Calfon M, et al. An integrated stress response regulates amino acid metabolism and resistance to oxidative stress. Mol Cell. 2003;11(3):619–33. 10.1016/s1097-2765(03)00105-9. 12667446 10.1016/s1097-2765(03)00105-9

[70] Hocsak E, Szabo V, Kalman N, Antus C, Cseh A, Sumegi K, et al. PARP inhibition protects mitochondria and reduces ROS production via PARP-1-ATF4-MKP-1-MAPK retrograde pathway. Free Radic Biol Med. 2017;108:770–84. 10.1016/j.freeradbiomed.2017.04.018. 28457938 10.1016/j.freeradbiomed.2017.04.018

[71] Li W, Li W, Zhang W, Wang H, Yu L, Yang P, et al. Exogenous melatonin ameliorates steroid-induced osteonecrosis of the femoral head by modulating ferroptosis through GDF15-mediated signaling. Stem Cell Res Ther. 2023;14(1):171. 10.1186/s13287-023-03371-y. 37400902 10.1186/s13287-023-03371-y PMC10318673

[72] Nguyen TT, Wei S, Nguyen TH, Jo Y, Zhang Y, Park W, et al. Mitochondria-associated programmed cell death as a therapeutic target for age-related disease. Exp Mol Med. 2023;55(8):1595–619. 10.1038/s12276-023-01046-5. 37612409 10.1038/s12276-023-01046-5 PMC10474116

[73] Ackermann K, Bonaterra GA, Kinscherf R, Schwarz A. Growth differentiation factor-15 regulates oxLDL-induced lipid homeostasis and autophagy in human macrophages. Atherosclerosis. 2019;281:128–36. 10.1016/j.atherosclerosis.2018.12.009. 30658188 10.1016/j.atherosclerosis.2018.12.009

[74] Myojin Y, Hikita H, Sugiyama M, Sasaki Y, Fukumoto K, Sakane S, et al. Hepatic Stellate Cells in Hepatocellular Carcinoma Promote Tumor Growth Via Growth Differentiation Factor 15 Production. Gastroenterology. 2021;160(5):1741-1754.e1716. 10.1053/j.gastro.2020.12.015. 33346004 10.1053/j.gastro.2020.12.015

[75] Shao Y, Jia H, Huang L, Li S, Wang C, Aikemu B, et al. An original ferroptosis-related gene signature effectively predicts the prognosis and clinical status for colorectal cancer patients. Front Oncol. 2021;11:711776. 10.3389/fonc.2021.711776. 34249766 10.3389/fonc.2021.711776 PMC8264263

[76] Jiang M, Jia K, Wang L, Li W, Chen B, Liu Y, et al. Alterations of DNA damage response pathway: biomarker and therapeutic strategy for cancer immunotherapy. Acta Pharm Sin B. 2021;11(10):2983–94. 10.1016/j.apsb.2021.01.003. 34729299 10.1016/j.apsb.2021.01.003 PMC8546664

[77] Mustofa MK, Tanoue Y, Tateishi C, Vaziri C, Tateishi S. Roles of Chk2/CHEK2 in guarding against environmentally induced DNA damage and replication-stress. Environ Mol Mutagen. 2020;61(7):730–5. 10.1002/em.22397. 32578892 10.1002/em.22397

[78] Li S, Wang L, Wang Y, Zhang C, Hong Z, Han Z. The synthetic lethality of targeting cell cycle checkpoints and PARPs in cancer treatment. J Hematol Oncol. 2022;15(1):147. 10.1186/s13045-022-01360-x. 36253861 10.1186/s13045-022-01360-x PMC9578258

[79] Huff LA, Yan S, Clemens MG. Mechanisms of Ataxia Telangiectasia Mutated (ATM) Control in the DNA Damage Response to Oxidative Stress, Epigenetic Regulation, and Persistent Innate Immune Suppression Following Sepsis. Antioxidants (Basel). 2021;10(7). 10.3390/antiox10071146. 10.3390/antiox10071146 PMC8301080 34356379

[80] González-Quiroz M, Blondel A, Sagredo A, Hetz C, Chevet E, Pedeux R. When endoplasmic reticulum proteostasis meets the DNA damage response. Trends Cell Biol. 2020;30(11):881–91. 10.1016/j.tcb.2020.09.002. 33036871 10.1016/j.tcb.2020.09.002

