# Supplementary material for: PARP inhibitor BMN673 triggers PARylation-mediated ATF4-GDF15 pathway to drive autophagy and ferroptosis in ataxia telangiectasia mutated gene-deficient colorectal cancer cells
Source: Mol Biomed. 2025 Nov 21;6:113. doi: 10.1186/s43556-025-00356-6 (PMC12635010; doi:10.1186/s43556-025-00356-6)
Supplement: Supplementary file 1 — Supplementary Material 1. [file 43556_2025_356_MOESM1_ESM.docx]

**PARP inhibitor BMN673 triggers PARylation-mediated ATF4-GDF15 pathway to drive autophagy and ferroptosis in ataxia telangiectasia mutated gene-deficient colorectal cancer cells**

**Junqi Xiang^1, 2*^, Jie Xu^3*^, Hui Fan^1*^, Qian Chen^1^, Yiting Lu^1^, Xinyan Wan^1^, Ying Jiang^1^, Xia Zhang^1^, Chundong Zhang^1^, Qingyuan Liu^4^, Degang Ding^4^**
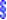
**, Yunlong Lei^1, 2^**
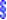


^1^ Department of Biochemistry and Molecular Biology, and Molecular Medicine and Cancer Research Center, College of Basic Medical Sciences, Chongqing Medical University, Chongqing 400016, China

^2^ Tianfu Jincheng Laboratory, Chengdu, 610093, China

^3^ The Center for Clinical Molecular Medical Detection, First Affiliated Hospital of Chongqing Medical University, Chongqing 400016, China

^4^ Department of Urology, Henan Provincial People’s Hospital, Zhengzhou 450003, China

* These authors contributed equally to this work.


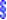
 Correspondence to:

Prof. Yunlong Lei, Department of Biochemistry and Molecular Biology, and Molecular Medicine and Cancer Research Center, College of Basic Medical Sciences, Chongqing Medical University, Chongqing 400016, P. R. China.

Email: [leiyunglong@cqmu.edu.cn](mailto:leiyunglong@cqmu.edu.cn)

Phone: +86 13648323625

Prof. Degang Ding, Department of Urology, Henan Provincial People’s Hospital, Zhengzhou 450003, China

Email: 13503848199@163.com

Phone: +86 13503848199

**Supplementary Materials and methods**

**1. Western blotting**

Cells were lysed with RIPA buffer (Beyotime) supplemented with the protease inhibitor cocktail (Sigma) for 30 min on ice. The protein lysate was sonicated on ice and centrifuged at 14, 000 rpm at 4℃ for 15 min. Protein concentrations were determined using the Quick Start^TM^ Bradford 1x dye reagent (500-0205, Bio Rad) and the protein lysates were boiled with the loading buffer. Proteins were separated on the SDS-PAGE gel (Bio-Rad) and transferred to the PVDF membranes (Millipore, United States), where they were blocked with 5% skimmed milk. The membranes were incubated with the corresponding primary antibodies at 4℃ overnight and HRP-conjugated secondary antibodies for 1 h at room temperature. Specific protein bands were visualized with a ChemiDoc image detector (Bio-Rad). Buffers used to dilute the primary antibodies and secondary antibodies were Tris-buffered saline with Tween 20 (TBST) and 5% skimmed milk respectively. The information of the primary and secondary antibodies and their working concentrations used in our experiments were detailed in **Supplementary Table 3**.

**2. Flow cytometric analysis**

For cell cycle analysis, cells were washed with ice-cold phosphate-buffered saline (PBS), and fixed with 70% ethanol. The cells were then treated with 50 μg/ml of RNase A and 50 μg/ml of propidium iodide for 30 min at room temperature. The mitochondrial membrane potential (MMP) was analyzed using the JC-1 assay kit (KeyGEN BioTECH, KGA603). All of these experiments were performed according to the corresponding manufacturer’s instructions. At least 10, 000 live cells were subjected to flow cytometric analysis on the FACScan fow cy-tometer (BD Biosciences). Experimental data were analyzed using FlowJo software.

**3. Transfection, stable ATM knockdown and overexpression cell generation**

The ATM wild-type, mutant plasmids (ATM*^D1853N^*) and shATM-DNA primers were purchased from Sangon BioEngineering (Shanghai) Co., Ltd. The shRNA target sequences were as follows: non-targeting control (NC), 5’- CAACAAGATGAAGAGCACCAA-3’; shATM-1, 5’- GGTGCTATTTACGGAGCTG-3’; and shATM-2, 5’- GCAACATACTACTCAAAGA-3’. The fragments were annealed and mixed with the digested plasmid, and the T4 DNA Ligase enzyme was added to incubate the recombination. Then the product was transformed by Stbl3 competent cell sent to the TSINGKE Co. Ltd for sequence detection, and finally the plasmid was extracted. The lentivirus particles were respectively co-transfected into 293T cells to produce recombinant lentiviral particles. After 48 h, viral supernatants were collected and infected the target cells. Forty-eight hours after lentivirus infection, cells were selected with puromycin (1μg/ml) for about 72 h to generate the stable ATM knockdown and overexpression cells. Stably transfected clones were validated by immunoblotting analysis. Other transfection experiments were conducted by using Lipofectamine 3000 (Thermo, L3000001) following the manufacturer’s instructions. The sequences of the siRNAs used are listed in **Supplementary Table 4**.

**4. Cell viability and colony formation assay**

Cell viability was assessed by CCK8 assay. Briefly, cells were seeded in 96-well plates overnight and subjected to different treatments. After treatment, CCK8 (Bimake, B34302) reagents were added to each well for 1 h at 37℃. The absorbance value was then determined at 450 nm with a spectrometer. For colony formation assay, after treatment, cells were cultured in 12-well plates overnight and subjected to different treatments. After 14 days, cells were fixed with 4% paraformaldehyde (Sigma) for 30 min and stained with Crystal Violet for another 30 min, and then the colonies were washed three times and taken photos.

**5. Transmission electron microscopy**

After 48 h treatment with BMN673 and chloroquine (CQ), SK-CO-1 and shATM-RKO cells were fixed in 4% glutaraldehyde (Sigma). The Cells were then postfixed, dehydrated, and embedded. The ultrathin sections were prepared using a sorvall MT5000 microtome. Then, the sections were stained by lead citrate and/or 1% uranyl acetate and visualized by Philips EM420 electron microscopy.

**6. Immunoblotting and immunoprecipitation**

Cells were lysed with RIPA buffer (Beyotime) supplemented with protease inhibitor cocktail (Sigma) and then protein lysates were centrifuged and boiled with loading buffer. For immunoprecipitations, cells were lysed with IP lysis buffer (Beyotime) and incubated with 1 μg antibody overnight at 4℃. Next day, Sepharose protein A/protein G beads were added for 2 h. The immune-complexes were then centrifuged and washed 3 times using RIPA buffer. All lysates were quantified by the BCA Protein Assay (Thermo Fisher Scientific) and analyzed by SDS-PAGE.

**7. Immunofluorescence**

Cells were seeded in 24-well plates with coverslips overnight and subjected to different treatments. The treated cells were fixed with 4% paraformaldehyde (Sigma) for 30 min and then washed three times with PBS. Fixed cells were permeabilized with 0.5% Triton X-100 for 12 min and blocked with 1% BSA for 2 h at 37℃. For staining, cells were incubated with primary antibodies for 12 h at 4℃, followed by incubation with secondary antibodies for 2 h at 37℃. Finally, nuclei were stained with DAPI for 10 min and then washed three times with PBS. For autophagic flux studies, cells were transfected with GFP-RFP-LC3 for 24 h and subjected to different treatments. Images were captured using a confocal microscopy (Leica).

**8. Quantitative RT-PCR (qRT-PCR)**

Total RNA was extracted using Trizol reagent (Invitrogen) and reverse transcribed using Reverse Transcription PrimeScript 1st Stand cDNA Synthesis kit (TaKaRa, Otsu, Japan). qRT-PCRs were performed using quantitative PCR reagents SYBR PremixEx TaqTM (TaKaRa) following the manufacturer’s instructions. Levels of GAPDH were used as an internal control and fold-changes were analyzed using the 2^−ΔΔCt^ method. The qRT-PCR primer sequences were shown in **Supplementary Table 5**.

**9. RNA-sequencing Analysis**

For RNA-seq analysis, the control and BMN-673 treated SK-CO-1 cells were collected, total RNA was extracted, and sequencing was performed by BGI. Bowtie2 (2.3.4.3) was used to align the clean data to the reference gene set. RSEM (v1.3.1) software was used for gene expression quantification, and pheatmap (v1.0.8) was used to draw the cluster heatmap of gene expression in different samples. Differential gene detection was performed using DESeq2 (1.4.5) (or DEGseq or PoissnDis) with Q value≦0.05 or FDR≦0.001. To further explore the gene functions associated with phenotypic changes, we based on hypergeometric tests, use Phyper (https://en.wikipedia.org/wiki/Hypergeometric distribution) to GO (https://www.geneontology.org/) and KEGG genetic variations (https://www.genome.jp/kegg/) enrichment analysis, Q value≦0.05 was used as the threshold, and significant enrichment in candidate genes was defined as meeting this condition. A cross-analysis between differentially expressed genes and iron death database (FerrDb website -http://www.zhounan.org/ferrdb/) was performed to filter out common genetic variations.

**10. Metabolomics analysis**

For metabolomics, 6 × 10^6^ SK-CO-1 cells and shATM-RKO cells per group are collected and frozen for subsequent analysis. Briefly, the cells were washed twice with cold PBS and incubated with pre-chilled 80% methanol (−80℃) for 1 h. Then, the cells were scraped in 80% methanol on dry ice and centrifuged for 5 min. The extracted metabolites in the supernatant were dried using a lyophilizer and the protein concentrations of pellets were measured for normalization. The dried metabolites were dissolved in 80% methanol and analyzed by LC–MS/MS. We used the TSQ Quantiva Triple Quadrupole Mass Spectrometer (Thermo Fisher Scientific, Waltham, MA, USA) with positive/negative ion switching for the quantitative analysis of targeted metabolites. The Q-Exactive Mass Spectrometer (Thermo Fisher Scientific, Waltham, MA, USA) was chosen for untargeted metabolites profiling. Metabolites were identified based on accurate ion masses and MS/MS fragments. Relative quantitation of metabolites was analyzed with TraceFinder 3.2 (Thermo Fisher Scientific, Waltham, MA, USA).

**11.** **Mitochondrial mass determination**

The mitochondrial mass was analyzed by MitoTracker Green (Beyotime, C1048) staining. Cells were seeded in 96-well plates overnight and subjected to different treatments. Cells were then incubated with MitoTracker Green according to the manufacturer’s instructions, followed by measuring the cellular fluorescent intensity using a Fluorescence Microplate Reader (Infinite TM M200, TECAN).

**12. Bioenergetic Analysis of Oxygen Consumption Rate (OCR) and Extracellular Acidification Rate (ECAR)**

OCR and ECAR were measured using the XF Cell Mito Stress Test and XF Glycolysis Stress test, respectively, on an Extracellular Flux Analyzer XFp (Agilent Technologies, Santa Clara, CA, USA). ATP production was measured by calculating the change in OCR using Cell Mito Stress test: (last rate measurement before Oligomycin injection) - (Minimum rate measurement after Oligomycin injection). Cells were seeded in 96-well plates at a density of 2 × 10^4^ cells/well with DMEM medium supplemented with 10% FBS and 0.1 mg/mL kanamycin. Agilent Seahorse XF Base Medium with glucose (final concentration: 10 mM), sodium Pyruvate (final concentration: 1 mM), and L-glutamine (final concentration: 2 mM) were used for the analysis medium. Oligomycin (final concentration: 2 μM), FCCP (final concentration: 2 μM), and Rotenone antimycin A (final concentration: 0.5 μM) were used to perform Cell Mito Stress Test. Glucose (final concentration: 10 mM), Oligomycin (final concentration: 1 μM), and 2-Deoxy-D-glucose (2-DG) (final concentration: 50 mM) were used in Glycolysis Stress test. We incubated the cartridge with a O_2_-free and CO_2_-free atmosphere at 37℃ to avoid the risk of cell reoxygenation during the Seahorse analysis.

**13. Measurement of glutathione (GSH)**

Total glutathione in cell lysates was measured with GSH detection kit (Beyotime, S0052) according to the manufacturer’s instruction. Briefly, GSH (1 mM) and CPMNSs (50 μg ml^−1^) mixture reacted for 3 hours; then, DTNB (0.5 mM) was added to detect the residual GSH, and H_2_O_2_ (1 mM) as a GSH depletion inducer was also evaluated. The GSH amount was detected by DTNB at the absorption of 412 nm.

**14. Measurement of malondialdehyde (MDA)**

The cell lysates were sonicated, and then centrifuged at 12, 000×g for 10 min at 4℃ to collect the supernatant. Total protein content was determined by using the Bradford assay. MDA levels were measured with MDA detection kit (Beyotime, S0131) according to the manufacturer’s instruction and detected at the absorption of 532 nm.

**Supplementary Figures**


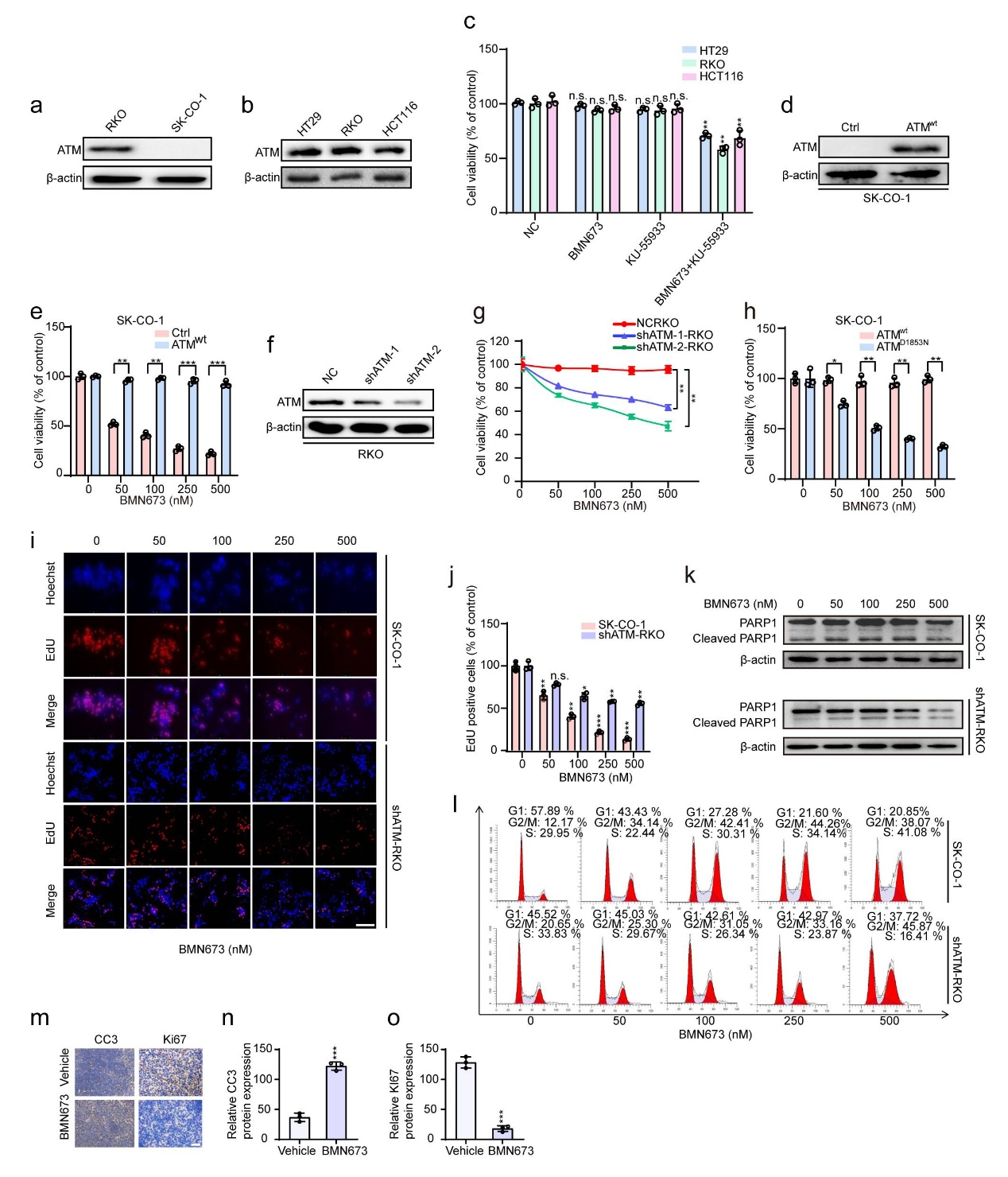
**Supplementary Figure 1. BMN673 exerts anti-cancer effects in ATM-deficient or mutated colorectal cancer cells.** (a) Immunoblot analysis of ATM expression in SK-CO-1 cells. (b) Immunoblot analysis of ATM expression in HT29, RKO and HCT116 cells. (c) HT-29, RKO and HCT-116 cells were treated with BMN673 (50 nM) alone or in combination with 10 μM KU-55933 for 48 h. The cell viability was examined by CCK8 assay. (d) Immunoblot analysis of ATM expression in SK-CO-1 cells with or without transfection of a wild-type ATM plasmid. (e) SK-CO-1 cells with or without transfection of a wild-type ATM plasmid were treated the indicated concentrations of BMN673 for 48 h. The cell viability was examined by CCK8 assay. (f) Immunoblot analysis of ATM expression in non-targeting control（NC）RKO and shATM-RKO cells. (g) CCK8 assay of NC RKO and shATM-RKO cells treated with the indicated concentrations of BMN673 for 48 h. (h) SK-CO-1 cells with transfection of a wild-type ATM plasmid or ATM*^D1853N^* plasmid were treated with indicated concentrations of BMN673 for 48 h. The cell viability was examined by CCK8 assay. (i) SK-CO-1 and shATM-RKO cells treated with the indicated concentration of BMN673 for 48 h, and the EdU incorporation (j) was quantitated. Scale bar, 100 μm. (k) Immunoblot analysis of PARP1 and Cleaved PARP1 expression in SK-CO-1 and shATM-RKO cells treated with the indicated concentrations of BMN673 for 48 h. (l) Cell cycle distribution. SK-CO-1 and shATM-RKO cells seeded on six-well plates were treated with the indicated concentrations of BMN673 for 48 h. Cells were harvested and analyzed by flow cytometry. (m) Immunohistochemical staining of CC3 and Ki67 in SK-CO-1 xenografts collected from vehicle or BMN673-treated mice. Scale bar, 50 μm. (n, o) Quantitation of CC3 and Ki67 expression. Data are means ± s.d. and are representative of 3 independent experiments. *, P < 0.05, **, P < 0.01, ***, P < 0.001.


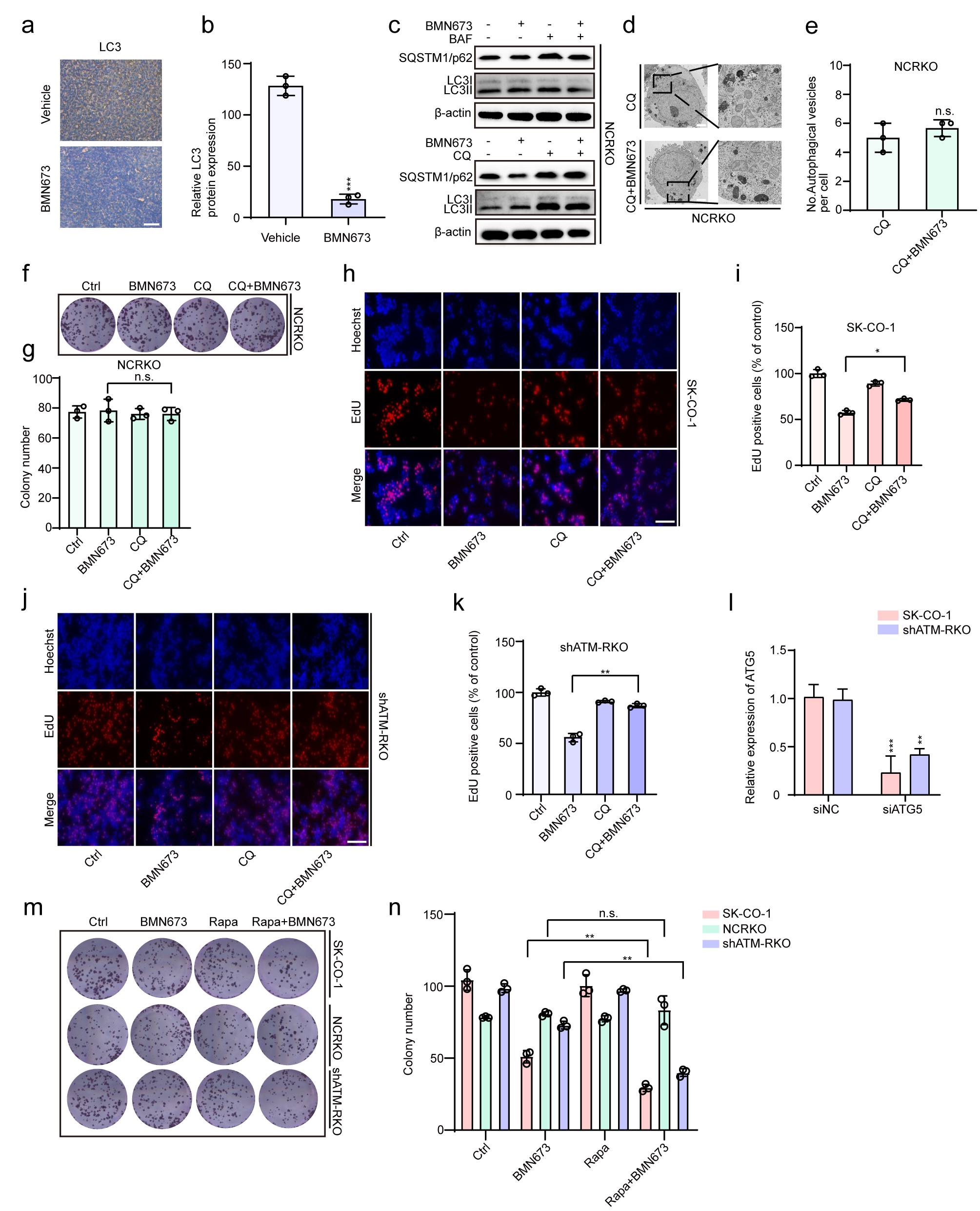
**Supplementary Figure 2. Autophagy potentiates BMN673-induced anticancer effects.** (a-b) Immunohistochemical staining of LC3 in SK-CO-1 xenografts collected from vehicle or BMN673-treated mice. Quantitation of LC3 expression (b) was shown. Scale bar, 50 μm. (c) NCRKO were treated with BMN673 (50 nM) alone or in combination with 10 μM CQ or 100 nM BAF for 48 h. The expressions of LC3B and P62 were examined by immunoblotting. (d, e) Autophagic vesicles were detected by transmission electron microscope in NCRKO cells treated with or without 50 nM BMN673 in combination with 10 μM CQ for 48 h. Scale bar, 1 μm. (f-g) Cell proliferation rate was analyzed by clone formation assay. NCRKO cells were treated with 50 nM BMN673 in the presence or absence of 10 μM CQ for 48 h, after treatment, cells were seeded into 12-well plates for two weeks and colony numbers (g) were quantified. (h-k) EdU assay of SK-CO-1 (h, i) and shATM-RKO (j, k) cells treated with 50 nM BMN673 in the presence or absence of 10 μM CQ for 48 h. The EdU incorporation was quantitated. Scale bar, 10 μm. (l) qRT-PCR was conducted in SK-CO-1 and shATM-RKO cells with siNC or siATG5 for 24 h to determine the transcription of ATG5. (m-n) Cell proliferation rate was analyzed by clone formation assay. SK-CO-1, NCRKO and shATM-RKO cells were treated with 50 nM BMN673 in the presence or absence of 10 μM Rapa for 48 h, after treatment, cells were seeded into 12-well plates for two weeks and colony numbers (n) were quantified. Data are means ± s.d. and are representative of 3 independent experiments. *, P < 0.05, **, P < 0.01, ***, P < 0.001.


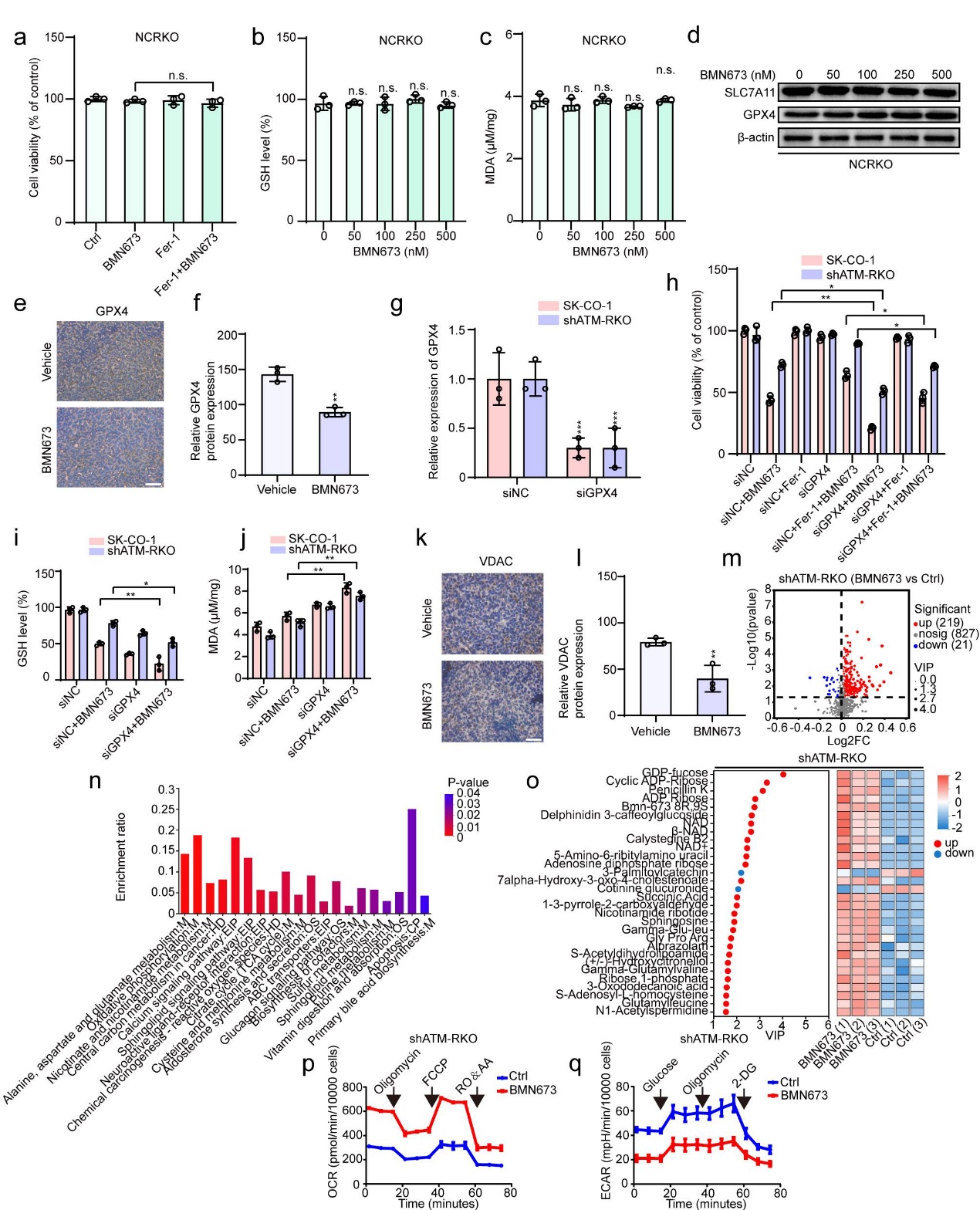
**Supplementary Figure 3. GPX4 downregulation promotes ferroptosis and oxidative phosphorylation is enhanced in ATM-deficient colorectal cancer cells treated with BMN673.** (a) NCRKO cells were treated with 50 nM BMN673 alone or in combination with 1μM Fer-1 for 48 h. The cell viability was examined by CCK8 assay. (b, c) GSH level (b) and MDA content (c) in NCRKO cells following indicated concentrations of BMN673 treatment for 48 h were determined. (d) Immunoblot analysis of SLC7A11 and GPX4 expression in NCRKO cells treated with the indicated concentrations of BMN673 for 48 h. (e-f) Immunohistochemical staining of GPX4 in SK-CO-1 xenografts collected from vehicle or BMN673-treated mice. Quantitation of GPX4 expression (f) was shown. Scale bar, 50 μm. (g) qRT-PCR was conducted in SK-CO-1 and shATM-RKO cells transfected with siNC or siGPX4 for 24 h to determine the transcription of GPX4. (h) CCK8 assay of SK-CO-1 and shATM-RKO cells treated with 1 μM Fer-1, followed by treatment with or without 50 nM BMN673 in the presence or absence of siGPX4 for another 48 h. (i, j) GSH level (i) and MDA content (j) in SK-CO-1 and shATM-RKO cells transfected with siGPX4 alone or in combination with 50 nM BMN673 for 48 h. (k-l) Immunohistochemical staining of VDAC in SK-CO-1 xenografts collected from vehicle or BMN673-treated mice. Quantitation of VDAC expression (l) was shown. Scale bar, 50 μm. (m-q) Differential metabolites were examined by metabolomics analysis in SK-CO-1 cells and shATM-RKO cells treated with 50 nM BMN673 for 48 h. (m) Volcano plot of all DAMs; (n) KEGG enrichment analysis; (o) VIP analysis; (p, q) OCR and ECAR in shATM-RKO cells treated with 50 nM BMN673 for 48 h was measured. Data are means ± s.d. and are representative of 3 independent experiments. *, P < 0.05, **, P < 0.01, ***, P < 0.001.


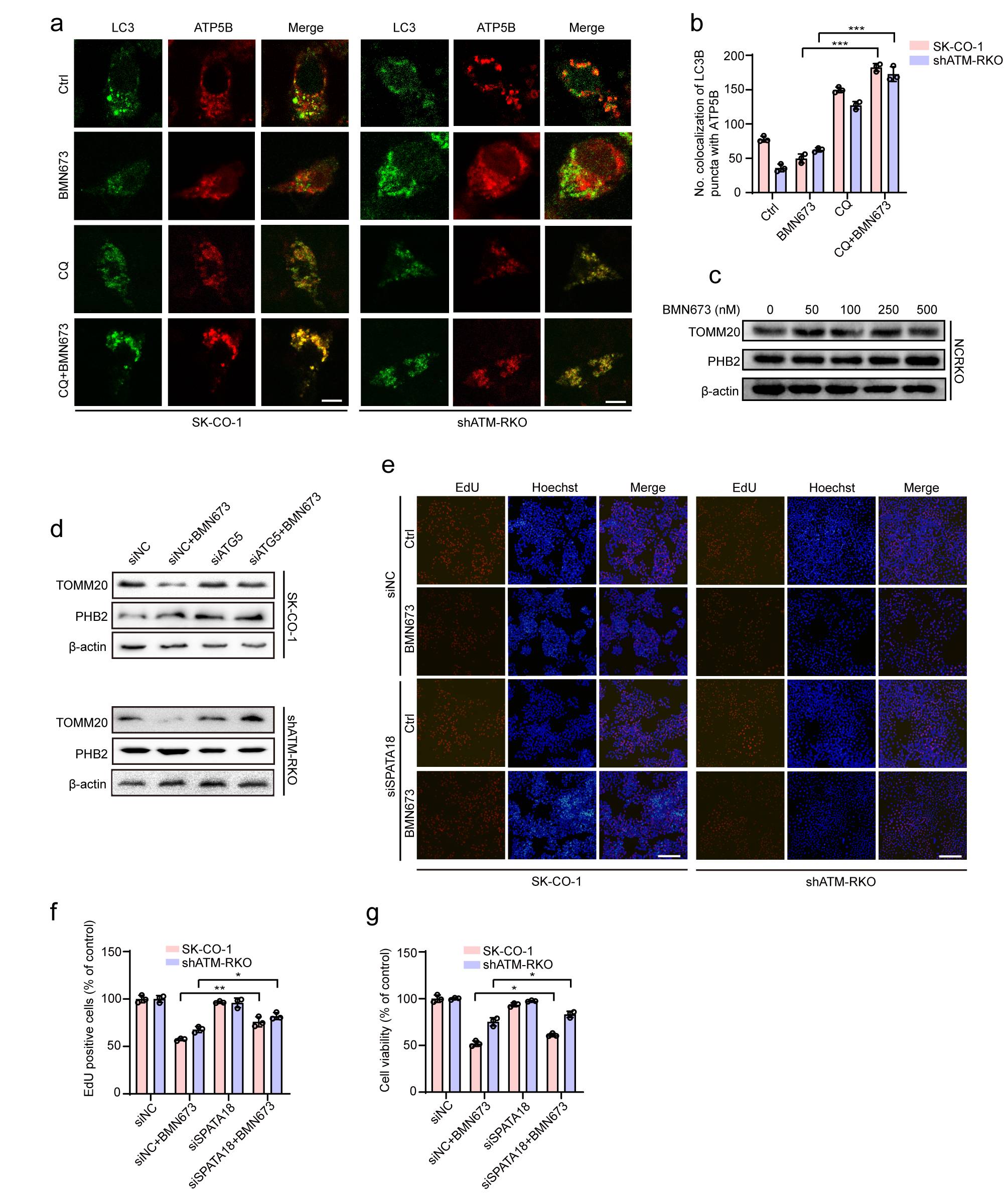
**Supplementary Figure 4. BMN673 promotes LC3-mediated conventional mitophagy and SPATA18 downregulation partially restores cell viability and proliferation in BMN673-treated ATM-deficient colorectal cancer cells.** (a, b) Immunofluorescence analysis of the co-localization of endogenous LC3B and ATP5B in SK-CO-1 and shATM-RKO cells treated with vehicle, BMN673 (50 nM), CQ (10 μM), or in combination for 48 h. Scale bar, 10 μm. (c) Immunoblot analysis of TOMM20 and PHB2 expression in NCRKO cells treated with the indicated concentrations of BMN673 for 48 h. (d) Immunoblot analysis of TOMM20 and PHB2 expression in SK-CO-1 and shATM-RKO cells transfected with siNC or siATG5 24 h, followed by treatment with or without 50 nM BMN673 for another 48 h. (e-g) SK-CO-1 and shATM-RKO cells transfected with siNC or siSPATA18 for 24 h, followed by treatment with or without 50 nM BMN673 for another 48 h. EdU assay of SK-CO-1 and shATM-RKO cells (e). Scale bar, 100 μm. (f) The EdU incorporation was quantitated. The cell viability was examined by CCK8 assay (g). Data are means ± s.d. and are representative of 3 independent experiments. *, P < 0.05, **, P < 0.01, ***, P < 0.001.


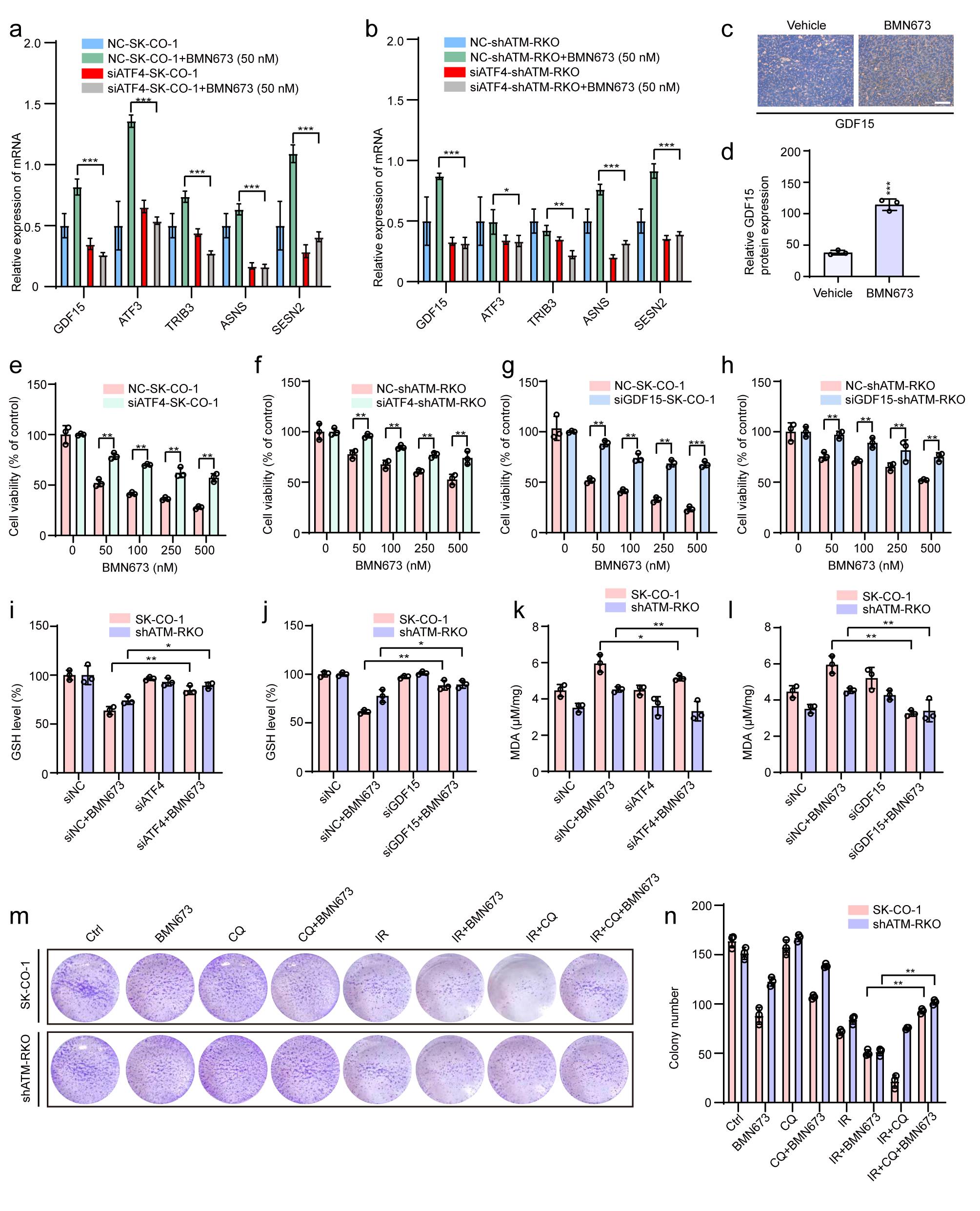
**Supplementary Figure 5. ATF4-GDF15 axis contributes to the anticancer effects of BMN673****, which sensitizes ATM-deficient colorectal cancer cells to RT.** (a, b) qRT-PCR was conducted in SK-CO-1 and shATM-RKO cells transfected with siNC or siATF4 for 24 h, followed by treatment with or without 50 nM BMN673 for another 24 h to determine the transcription level of GDF15, ATF3, TRIB3, ASNS and SESN2. (c, d) Immunohistochemical staining of GDF15 in SK-CO-1 xenografts collected from vehicle or BMN673-treated mice. Quantitation of GDF15 expression (d) was shown. Scale bar, 50 μm. (e-h) CCK8 assays of SK-CO-1 and shATM-RKO cells transfected with siNC, siATF4 or siGDF15 and treated with the indicated concentrations of BMN673 for 48 h. (i-l) GSH level (i, j) and MDA content (k, l) in SK-CO-1 and shATM-RKO cells transfected with siNC, siATF4 or siGDF15 for 24 h, followed by treatment with or without 100 nM BMN673 for another 48 h. (m, n) Clone formation assay of SK-CO-1 and shATM-RKO cells treated with 2 Gy IR, followed by treatment with or without 50 nM BMN673 in the presence or absence of 10 μM CQ for another 48 h, after treatment, cells were seeded into 12-well plates for two weeks and colony numbers (n) were quantified. Data are means ± s.d. and are representative of 3 independent experiments. *, P < 0.05, **, P < 0.01, ***, P < 0.001.


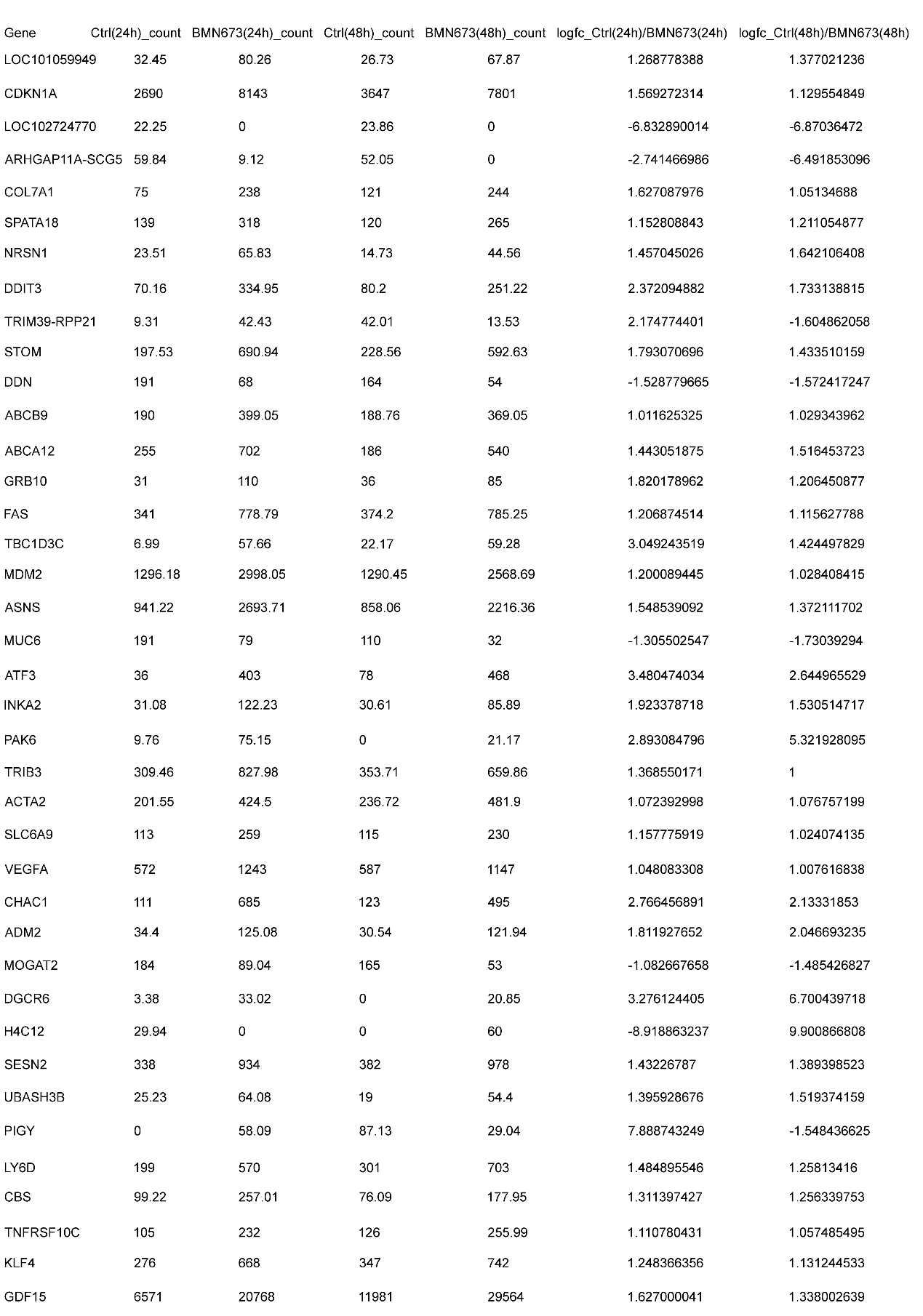
**Supplementary Table 1:** Gene features significantly differentially expressed in SK-CO-1 cells treated with BMN673


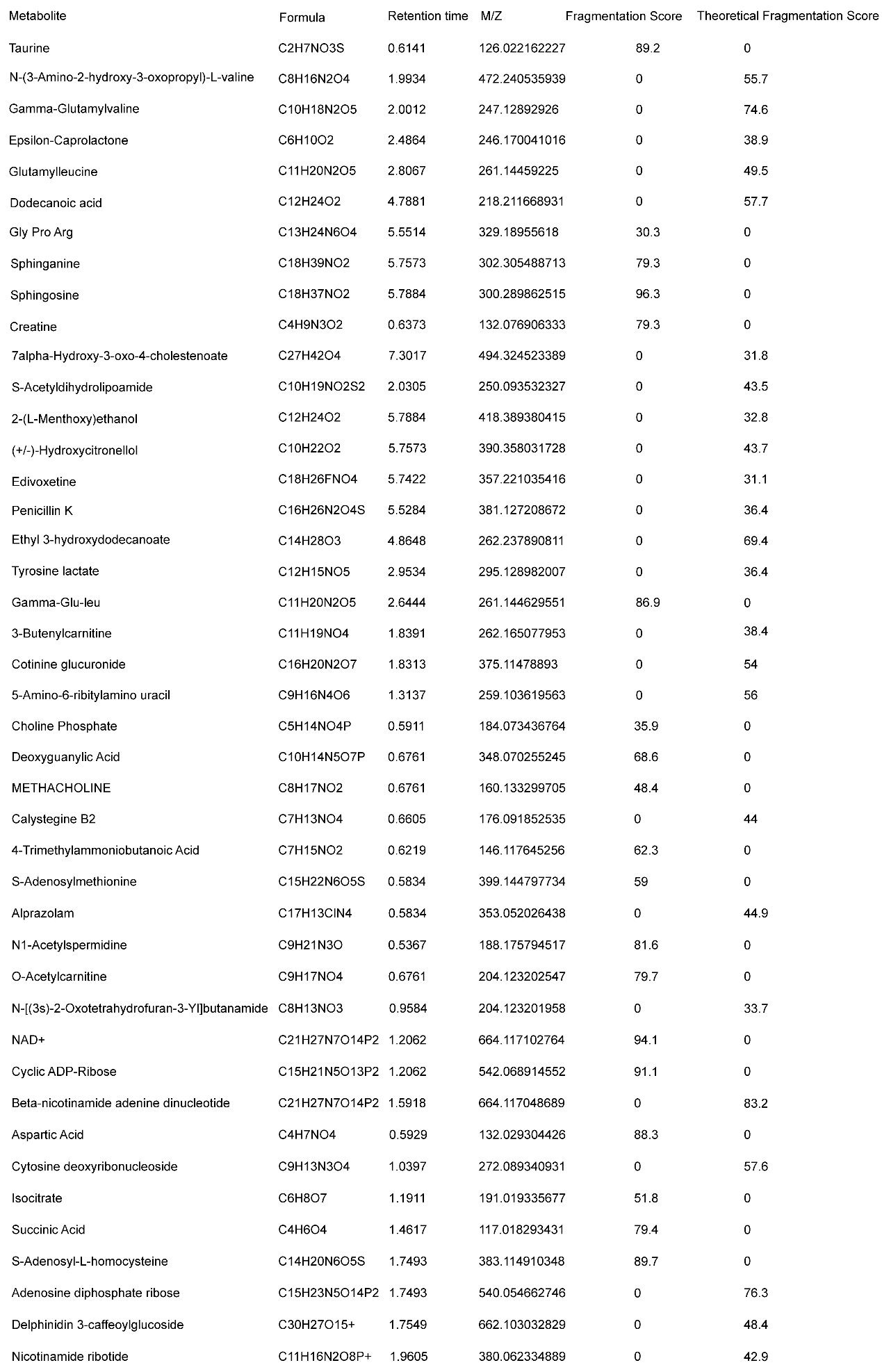
**Supplementary Table 2:** Metabolites features significantly differentially expressed in SK-CO-1 cells and shATM-RKO treated with BMN673


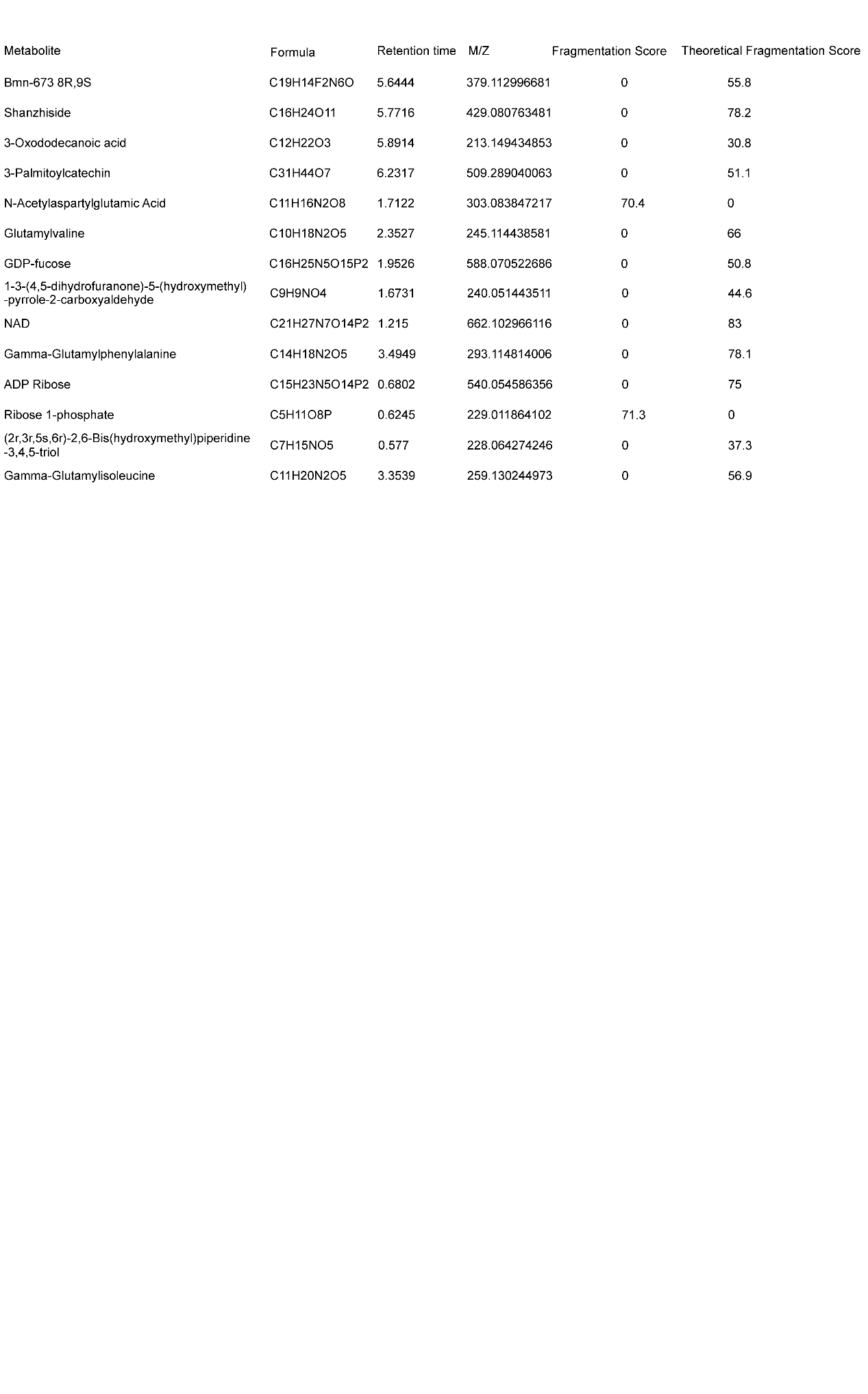
 **Supplementary Table 3:** Antibodies used in the present study

| **Name** | **Supplier** | **Cat no.** |
| --- | --- | --- |
| **ATM** | **Cell Signaling Technology** | **2873S** |
| **PARP1** | **Cell Signaling Technology** | **9542S** |
| **MAP1LC3B** | **Cell Signaling Technology** | **4108S** |
| **β-Actin** | **Abways Technology** | **AY0573** |
| **SLC7A11** | **Abcam** | **ab175186** |
| **GPX4** | **Abcam** | **ab125066** |
| **VDAC** | **Cell Signaling Technology** | **4661T** |
| **Ubiquitin** | **Cell Signaling Technology** | **3936T** |
| **PERK** | **Cell Signaling Technology** | **5683S** |
| **p-PERK** | **Affinity** | **DF7576** |
| **IRE1α** | **Cell Signaling Technology** | **3294T** |
| **p-IRE1α** | **Abcam** | **ab124945** |
| **CHOP** | **SAB** | **40744-2** |
| **ATF4** | **Cell Signaling Technology** | **11815S** |
| **GAPDH** | **Affinity** | **AF7021** |
| **GDF15** | **Abcam** | **ab180929** |
| **Histone H3** | **Abcam** | **ab1791** |
| **PAR** | **Sigma** | **AM-80** |
| **Caspase-3** | **Abcam** | **ab32351** |
| **Ki67** | **Cell Signaling Technology** | **9449T** |
| **Anti-Rabbit IgG** | **Proteintech** | **SA00001-2** |
| **Anti-Mouse IgG** | **Abcam** | **ab205719** |
| **Goat anti-Rabbit IgG (H+L) Cross-Adsorbed Secondary Antibody, Alexa Fluor 568** | **Thermo fisher** | **A-21069** |
| **Goat anti-Rabbit IgG (H+L) Cross-Adsorbed Secondary Antibody, Alexa Fluor 488** | **Thermo fisher** | **A-21222** |
| **SQSTM1/p62** | **Cell Signaling Technology** | **5114T** |
| **TOMM20** | **Cell Signaling Technology** | **42406T** |
| **ATP5B** | [**Santa Cruz Biotechnology**](https://www.scbt.com/zh/home) | **sc-74549** |
| **PHB2** | **Abiowell** | **AWA10697** |

**Supplementary Table 4:** List of Small Interference RNA Sequence

| **Gene name** | **sequences (5'-3')** |
| --- | --- |
| **ATF4** | **F: 5'-GCCUAGGUCUCUUAGAUGAUUTT-3'** |
| **GDF15** | **F: 5'-GCUACAAUCCCAUGGUGCUCAUUCATT-3'** |
| **ATG5** | **F: 5'-CCUGAACAGAAUCAUCCUUAATT****-3'** |
| **SPATA18** | **F: 5'-CAUGGAGAAUGACUGUGUCAUTT****-3'** |
| **GPX4** | **F: 5'-GCAACUGUCUGGAACUGCATT-3'** |

**Supplementary Table 5:** Oligonucleotides used in the present study

Primers used for RT-PCR analysis

| **Gene name** | **Primer sequence** |
| --- | --- |
| **GAPDH** | **F :5'-ACCTGACCTGCCGTCTAGAA-3'**  **R: 5'-TCCACCACCCTGTTGCTGTA-3'** |
| **SESN2** | **F :5'-AAGGACTACCTGCGGTTCG-3'**  **R: 5'-CGCCCAGAGGACATCAGTG-3'** |
| **SLC7A11** | **F :5'-TCTCCAAAGGAGGTTACCTGC-3'**  **R: 5'-AGACTCCCCTCAGTAAAGTGAC-3'** |
| **SPATA18** | **F :5'-GCAGGAAAAGCTAGACTTCTGG-3'**  **R: 5'-TGGCAACTTGCTCAATGAGTTC-3'** |
| **KLF4** | **F :5'-CAGCTTCACCTATCCGATCCG-3'**  **R: 5'-GACTCCCTGCCATAGAGGAGG-3'** |
| **GDF15** | **F :5'-CAACCAGAGCTGGGAAGATTCG-3'**  **R: 5'-CCCGAGAGATACGCAGGTGCA-3'** |
| **P21** | **F :5'-TGTCCGTCAGAACCCATGC-3'**  **R: 5'-AAAGTCGAAGTTCCATCGCTC-3'** |
| **ATF3** | **F :5'-GTGCCGAAACAAGAAGAAGG-3'**  **R: 5'-TCTGAGCCTTCAGTTCAGCA-3'** |
| **ATF4** | **F :5'-ATGACCGAAATGAGCTTCCTG-3'**  **R: 5'-GCTGGAGAACCCATGAGGT-3'** |
| **TRIB3** | **F :5'-AAGCGGTTGGAGTTGGATGAC-3'**  **R: 5'-CACGATCTGGAGCAGTAGGTG-3'** |
| **ASNS** | **F :5'-GGAAGACAGCCCCGATTTACT-3'**  **R: 5'-AGCACGAACTGTTGTAATGTCA-3'** |
| **GPX4** | **F :5'-GAGGCAAGACCGAAGTAAACTAC-3'**  **R: 5'-CCGAACTGGTTACACGGGAA-3'** |
